# Distinct expression of select and transcriptome-wide isolated 3’UTRs suggests critical roles in development and transition states

**DOI:** 10.1371/journal.pone.0250669

**Published:** 2021-05-05

**Authors:** Shaoyi Ji, Ze Yang, Leonardi Gozali, Thomas Kenney, Arif Kocabas, Carolyn Jinsook Park, Mary Hynes

**Affiliations:** 1 Dept. of Biology, Stanford University, Stanford, CA, United States of America; 2 Rockefeller University, New York, NY, United States of America; University of Alabama at Birmingham, UNITED STATES

## Abstract

Mature mRNA molecules are expected to be comprised of a 5’UTR, a 3’UTR and a coding region (CDS). Unexpectedly, however, there have been multiple recent reports of widespread differential expression of mRNA 3’UTRs and their cognate coding regions (CDS), reflecting the expression of isolated 3’UTRs (i3’UTRs); these i3’UTRs can be highly expressed, often in reciprocal patterns to their cognate CDS. As with other long non-coding (lncRNAs), isolated 3’UTRs are likely to play an important role in gene regulation, but little is known about the contexts in which they are deployed. To illuminate the functions of i3’UTRs, here we carry out *in vitro*, *in vivo* and *in silico* analyses of differential 3’UTR/CDS mRNA ratio usage across tissues, development and cell state changes both for a select list of developmentally important genes as well as by unbiased transcriptome-wide analyses. Across two developmental paradigms we find a distinct switch from high i3’UTR expression for stem cell related genes in proliferating cells to high CDS for these genes in newly differentiated cells. Unbiased transcriptome analysis across multiple gene sets shows that regardless of tissue, genes with high 3’UTR to CDS ratios belong predominantly to gene ontology categories related to cell-type specific functions. In contrast, the gene ontology categories of genes with low 3’UTR to CDS ratios are similar across tissues and relate to common cellular functions. We further show that, at least for some genes, traditional transcriptional start site genomic elements correspond to identified RNAseq 3’UTR peak regions, suggesting that some i3’UTRs may be generated by de novo transcription. Our results provide critical information from which detailed hypotheses for individual i3’UTRs can be tested, with a common theme that i3’UTRs appear poised to regulate cell-specific gene expression and state.

## Introduction

In contrast to the canonical view that an mRNA molecule is comprised of a 5’UTR, a 3’UTR and a coding region (CDS), we and others have found widespread stable expression of mRNA 3’UTRs in the absence of their cognate coding regions (CDS) [1–4] and CDS sequences in apparent absence of their 3’UTRs. These isolated 3’UTRs (i3’UTRs) can be highly expressed, often in reciprocal patterns to their cognate CDS, and are likely to play an important role in gene regulation, as do other long non-coding RNAs (lncRNA) [5–7]. With dual in situ hybridization (ISH)- we previously showed non-random differential 3’UTR to CDS expression in many tissues in the embryo and adult, and the 3’UTR to CDS ratio for a given gene is not fixed- for example young neurons express high levels of *Sox11* CDS compared to 3’UTR, but switch and then maintain high *Sox11* 3’UTR levels [2]. Thus, cells appear to expend high energy to maintain robust, non-random i3’UTR expression, suggesting that either the ratio of the 3’UTR to its cognate CDS (3’UTR/CDS), and/or the isolated 3’UTRs themselves may have important gene regulatory functions.

Historically there are a few reports of biological roles for 3’UTRs independent of their cognate CDS that were documented, when i3’UTR’s were intended as a control, but where their overexpression or deletion produced a biological effect [8–10]. More recently a specific role for a 3’UTR was described for axon growth and viability [11]. While most i3’UTRs are likely to have a biological role, as with long non-coding RNAs (lncRNAs), their actions/functions may be diverse and cell type contextual [12–16], mediated by binding to RNA, RNA binding proteins, and/or DNA and may be involved in functions ranging from the regulation of stem cell pluripotency to cancer progression [13, 17, 18]. For this reason, a thorough understanding of when, where and which mRNAs have a high tendency to show differential 3’UTR to cognate CDS expression across tissues and genes sets is useful and necessary to devise cogent hypotheses that can be developed and tested for any individual i3’UTR. Here we present *in vitro*, and *in vivo* data on the non-random and dynamic i3’UTR expression of select genes in progenitor cells, followed by *in silico*, unbiased transcriptome-wide analyses of open source RNAseq genes sets. The data suggest a strong proclivity for high 3’UTR to CDS expression in cells and for genes involved in transition states, during both proliferative and developmental maturation events. We also report that for some genes, traditional transcriptional start site genomic elements correspond to identified RNAseq 3’UTR peak regions, suggesting a potential mechanism for generation of i3’UTRs for those genes.

## Results

### Widespread use of differential 3’UTR to CDS usage and ISH specificity

**Fig 1:**
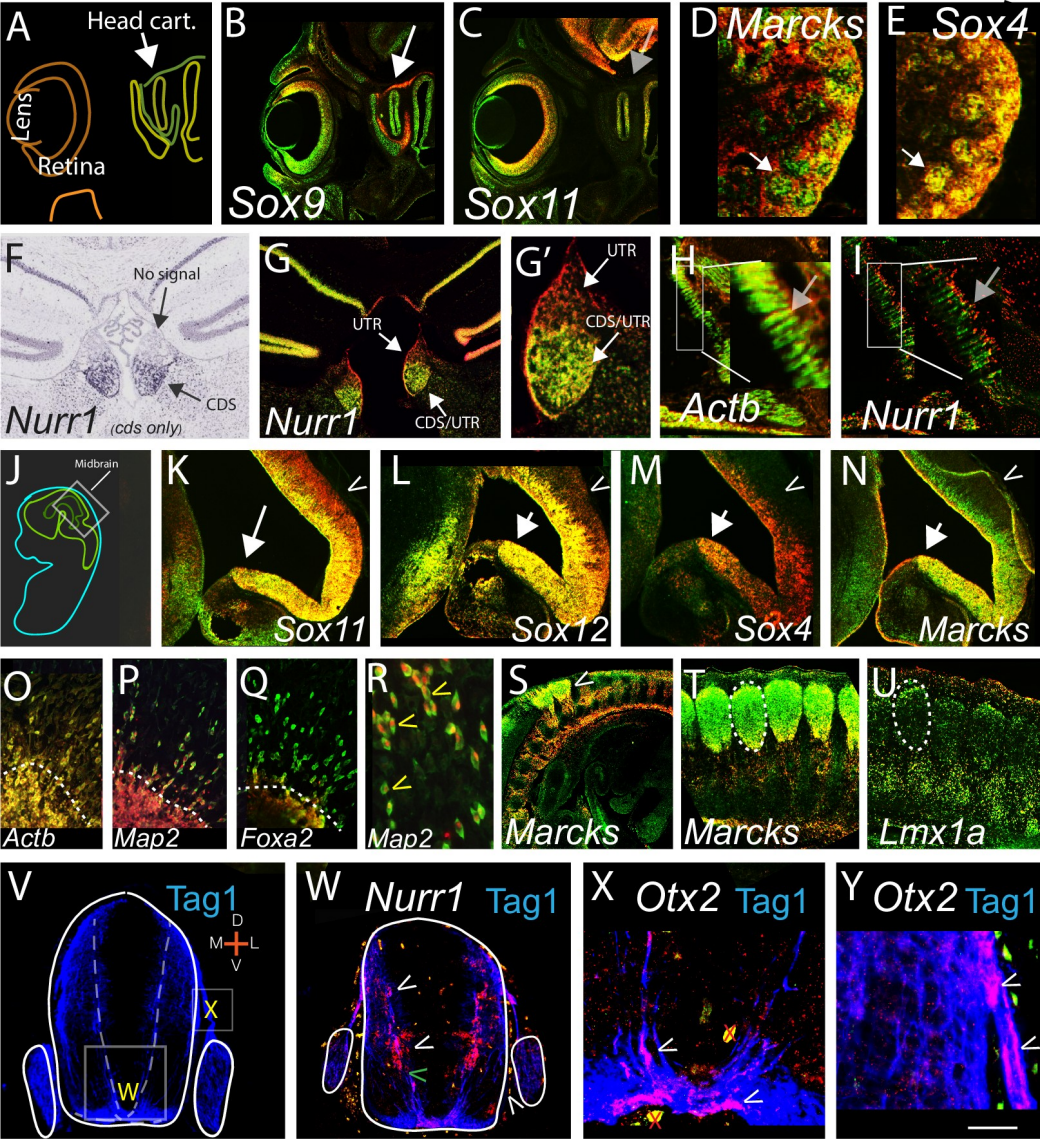
**Widespread, specific, differential 3’UTR (red)/cognate CDS (green) expression across mouse tissues and ages.** (A) Schematic of sagittal E15 eye and head cartilage region. (B,C) similar sections to A; probes for *Sox9* and *Sox11* detect unique signals (white arrow, signal, gray; no signal) (**unless specified, in all Fig 3‘UTR probe is red and CDS probe is green with both probes co-hybridized to same section**). (D, E and S1I, S1J Fig in S1 File) Kidney; *Marcks* shows high CDS in glomeruli, while *Sox4* shows equal 3’UTR/CDS in same region in adjacent section; white arrows. (F,G,G’); *Nurr1* in adult brain hybridized with Allen brain atlas single CDS probe (F) (https://developingmouse.brain-map.org/experiment/show/732) or in-house probes (G,G’). (G’) high power view of medial habenula showing high *Nurr1* 3’UTR throughout, CDS restricted ventrally. (I) *Nurr1* in E15 muscle shows distinctly high 3’UTR at muscle fiber tips while *Actb* (H*)* shows CDS with some 3’UTR throughout; (H,I; gray arrows; insets). (J) Schematic, sagittal E15 embryo. (K-N) Similar adjacent sections as J inset, showing *SoxC* family members and *Marcks*. Arrows show newly developing neurons, carrots show dorsal brain (additional genes in S1L, S1M Fig in S1 File). (O-Q) *Actb*,*Map2*,*Foxa2* 3’UTR/CDS in E15 neural explants *in vitro;* dotted line shows explant edge, cells above that are migrating on substrate. (R) High power showing *Map2* 3’UTR confined to the nucleus with the CDS in the cytoplasm in migrating cells. *Actb* also shows high 3’UTR nuclear signal in migrating neurons, while *Foxa2* (Q) does not. (n = 16 explants/probe; all probes showed replicate patterns). (S-Y) 3’UTR/CDS expression in and around developing axons; (S,T) In sagittal view *Marcks* shows high CDS in DRG cell bodies (dotted circle T) but high 3’UTR in axons (carrot; S). (U) *Lmx1a* in adjacent section shows no obvious axonal expression. (V-Y), E11 spinal cord-early developmental genes show high 3’UTR in or alongside Tag1+ axons (V). (W) *Nurr1* 3’UTR is highly expressed along axon path; carrots. (X,Y) *Otx2* in the axons; carrots. Both *Otx2* and *Nurr1* show robust CDS signal in other sections (G, and S1O Fig in S1 File or not shown). Scale bar, A-C; 600, D,E; 300 F,G; 450, G’ 200, H,I; 1200; inset 300 J; 1600 K-N; 300, O-Q 800, R; 300, S; 1500, T,U; 500, V,W; 220, X, 100, Y, 80 um. Red x’s in X are artifacts.

Fig 1 illustrates both the widespread use of differential 3’UTR to CDS expression across tissues and ages, as well as extensive controls for our dual in situ hybridization (ISH) procedure. To date we have examined > 60 cognate 3’UTR/CDS probe pairs across tissues and ages (herein, unpublished and [2]). Here, we document probe specificity, pattern replication and congruence with public data (Fig 1A–1G). First, when examining the 3’UTR to CDS expression of closely related *Sox* gene family members we observed overlapping, but distinct patterns for each probe pair (3’UTR and cognate CDS) in serial adjacent sections, such as for *Sox9* and *Sox11* in the developing head (Fig 1A–1C; arrows). In animals deleted for *Sox11*, all *Sox11* signal is absent, while *Sox2* and *Sox9* expression remains intact (S1A-S1H Fig and S1C vs S1D Fig in S1 File). In the developing mid-hindbrain, previous single probe ISH analyses shows *Sox* family member genes to have fairly ubiquitous, overlapping expression with undiscernible patterns (S1Q’ vs S1R Fig in S1 File; http://developingmouse.brain-map.org/experiment/show/100071526) [19, 20]. However, dual ISH with cognate 3’UTR/CDS probe pairs reveals dramatic differential expression for *Sox11*, *12* and 4, each with unique, and finely tuned distinct expression (Fig 1J–1N; S1L, S1M Fig in S1 File; *Sox11* shows higher CDS to 3’UTR in progenitor regions, equal CDS to 3’UTR in more mature neurons (Fig 1K; white arrow), and high 3’UTR to CDS in the most differentiated (dorsal) neurons (Fig 1K; carrot) with other *Sox* genes showing different patterns (Fig lL-1N; carrots). These data show that our probes and hybridization procedures result in specific signals. In addition, the use of cognate 3’UTR and CDS probes allow us to uncover previously unseen biological subtleties for closely related family members such as the *Sox* genes. These differences may be critical during important developmental windows.

We further compared in-house and custom RNAscope^r^ probes, finding indistinguishable hybridization patterns between the two (data not shown) and verified that co-hybridization with identical probes labeled with the two different fluorophores-digoxygenin or fluorescein- resulted in the essentially all positive cells appearing yellow, as expected from signal overlap (not shown). We did find slight non-overlap at tissues edges; thus, all data herein is from non-edge tissue regions. We verified that ISH signals replicated bilaterally, across sections and in multiple experiments and that dissimilar genes in serial sections show unique hybridization patterns, as in the developing kidney (Fig 1D–1E; arrows and S1I, S1J Fig in S1 File). We further compared our data to the widely used Allen Brain Atlas and find identical expression; as illustrated with the *Nurr1* CDS: both our and the Allen Brain CDS probes detect signal in ventral, but not dorsal, medial habenula (with Fig 1F, 1G and 1G’). Analysis of *Nurr1* 3’UTR expression shows that, in contrast to the restricted *Nurr1* CDS, the *Nurr1* 3’UTR is expressed widely across many tissues, including developing muscle, spinal cord and adult cerebral cortex, (Fig 1I; S1K, S1S Fig in S1 File). In the CNS *Nurr1* is known primarily for its role in dopaminergic (DA) neuron development but these data suggest a wider role in the nervous system, as well as a role in other tissues (see Discussion).

**Fig 2:**
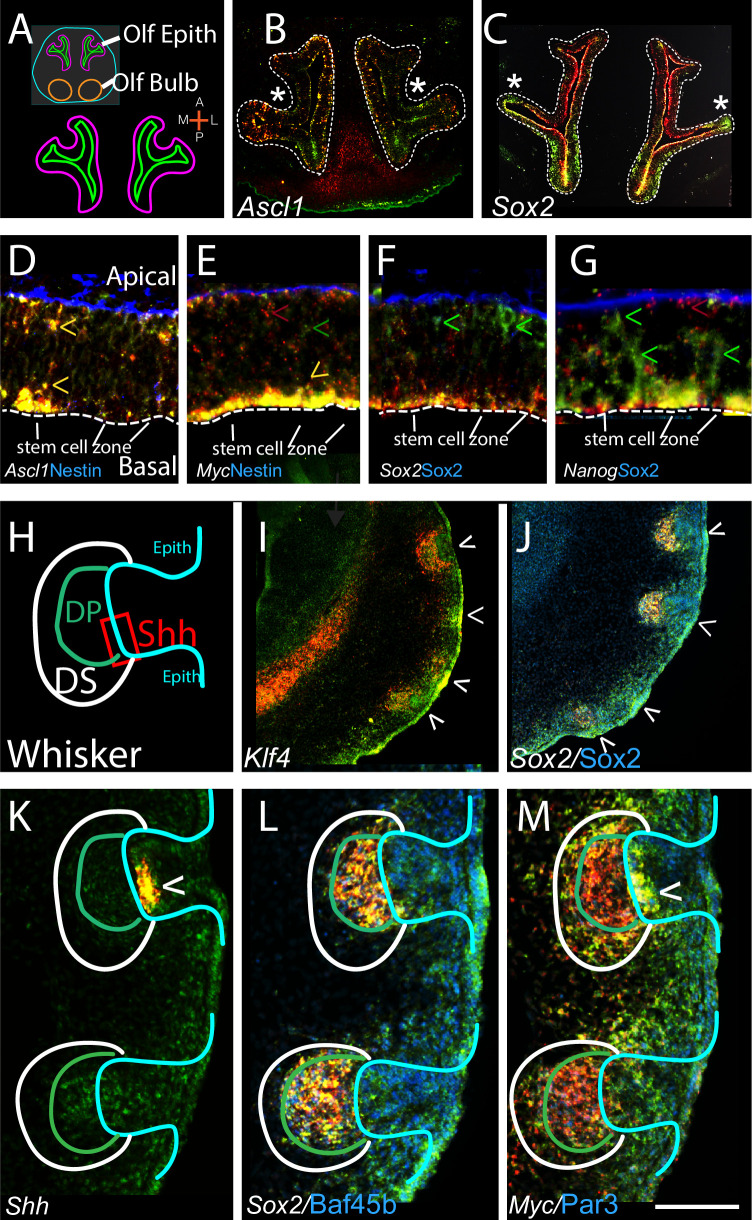
Progenitor cells show distinctive 3’UTR/CDS patterns. (A) Schematic showing horizontal view of E12 olfactory epithelium (OlfE). (B,C) similar sections of E12 OlfE with control pro-neural gene *Ascl1* and PpG gene *Sox2*. *Ascl1* is expressed in scattered cells, often with colocalized 3’UTR/CDS expression, while *Sox2* shows graded differential 3’UTR/CDS expression across OlfE regions. * denotes symmetrical patterns in contralateral OlfE for each gene, (additional genes in S2B, S2D-S2H Fig in S1 File). (D-G) higher power view of core PpG expression in OlfE SVZ; as labeled. Note that all PpGs (but not *Ascl1* (D)) show preferential localization of their 3’UTR sequences to the apical plate, labeled in D and same in E-G. Basal plate shows high Nestin (D,E) and Sox2 (F,G) protein and each CDS can also be colocalized to Nestin positive radial glia (seen at longer exposure) (green carrots). Apical stem cell zone is marked. For *Myc* both the 3’UTR and CDS are concentrated apically. For all other PPGs examined, *Sox2*, *Oct4*, *Klf4*, and *Nanog*, their 3’UTR expression is concentrated apically with the CDS signal both apical and extending along radial glia to the basal plate; carrots; F,G and S2 Fig in S1 File). (H) Schematic of whisker key anatomy. (I,J) low power views of four developing whiskers; top two with well-defined dermal papillae, bottom two- earlier condensates. Note that differential 3’UTR/CDS expression for all PpGs (I, J and S2M, S2N Fig in S1 File) is apparent and an early indicator of hair peg condensation. (K-M) higher power views showing differential 3’UTR/CDS for *Sox2* (L) and *Myc* (M) in anatomically distinct regions of the whisker. *Shh* (K) shows coincidental 3’UTR/CDS expression in an adjacent section (carrot). Other PgPs shown in S2I-S2L Fig in S1 File). Anatomical abbreviations are, Shh; *Shh* expression region, DP; dermal papilla, DS; dermal sheath (white). Scale bar, A; 500, B,C; 350, D-G; 150, H,K-M; 55, I,J; 350 um.

**Fig 3:**
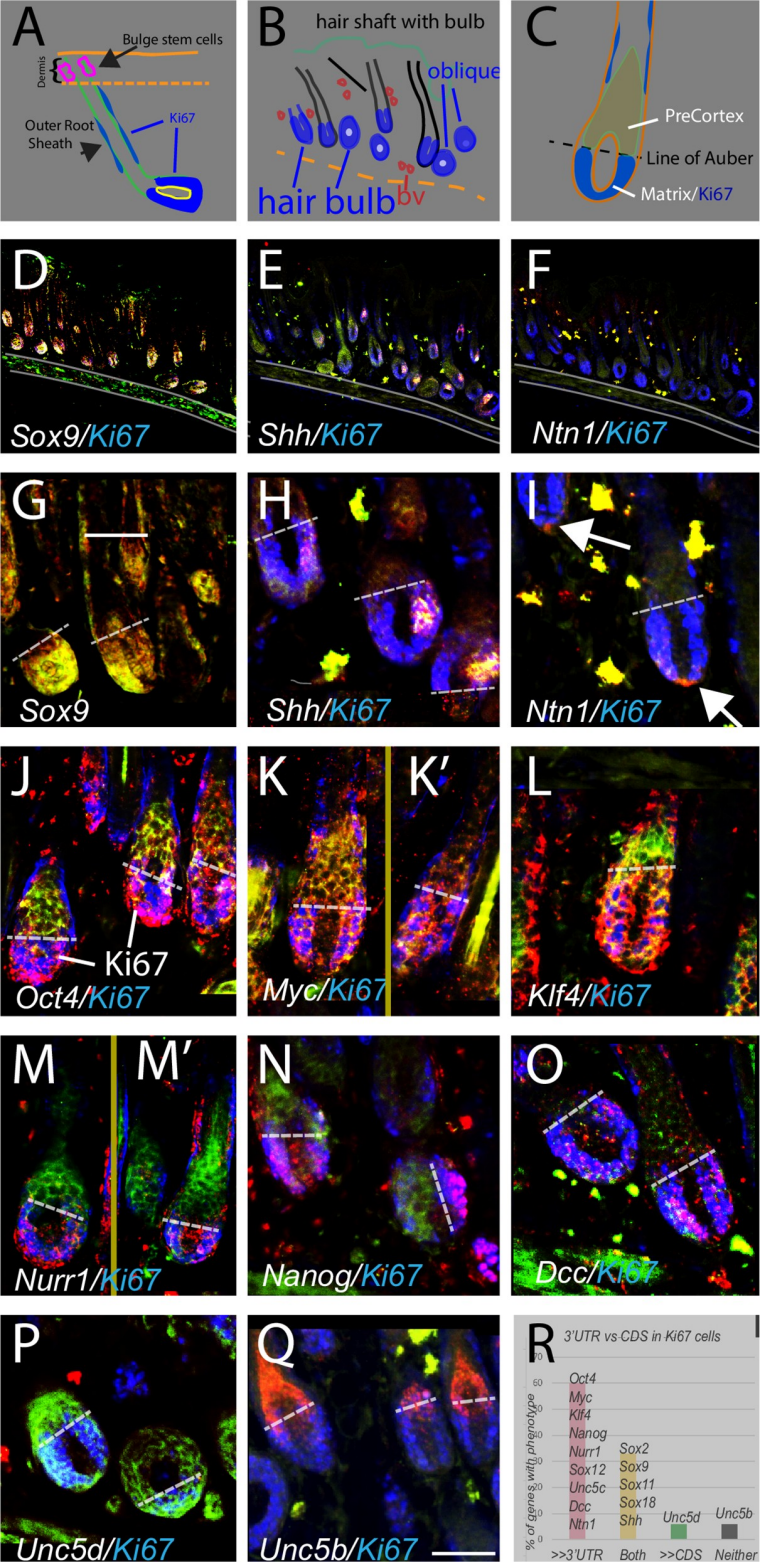
The 3’UTR/CDS ratio for PpG and other genes changes abruptly at the boundary between the Ki67+ proliferative zone and non-proliferative zone. (A) Key anatomy of skin hair follicle; stem cells located dorsally within dermis, outer root sheet (ORS) at the perimeter of the follicle, proliferating cells (Ki67+) in deep hair bulb and along ORS. (B) schematic of skin sections shown in (D-F), each section shows multiple hair follicles; oblique deep bulb follicles appear round. (C) coronal view of lower hair shaft showing location of Ki67+ matrix cells, and pre-cortex cell. The critical line of Auber (dotted line in C-Q) demarcates the boundary between proliferating Ki67+ cells and non-proliferating pre-cortex cells. (D-F) similar sections as B hybridized for *Sox9*, *Shh* and *Netrin-1 (Ntn1*). Note the different (and therefore specific) hybridization patterns for the different probes. (G-Q) higher power views of 1–3 hair bulbs for genes as labeled. Note abrupt shift from high 3’UTR in Ki67+ cells below line of Auber to Hi CDS above. (R) 60 percent of genes examined show high i3’UTR in Ki67+ cells with a switch at the line of Auber (n = 20–30 bulbs/gene). (K,K’, M, M”) cropped to remove hair shafts which can show high autofluorescence. Full picture of M can be seen in S3H Fig in S1 File. Second bulb in N is oblique (as in A) therefore line of Auber appears rotated. (O-Q); Netrin1 receptors. Like the PpGs, *DCC* (also known as a tumor suppressor gene) shows higher 3’UTR/CDS in Ki67+ cells (Fig 3O). In contrast *Unc5b* shows no expression in Ki67+ cells, with high 3’UTR above the line.Unc5d shows only CDS in the hair follicle even though there is high 3’UTR in the nearby blood vessels. *Ntn1* and *Unc5b* show high CDS and high 3’UTR in blood vessels (Fig 3I and 3Q; yellow signal). Scale bar, A; 160, B; 400, C; 220, D-F; 700, G-Q; 85 um.

**Fig 4:**
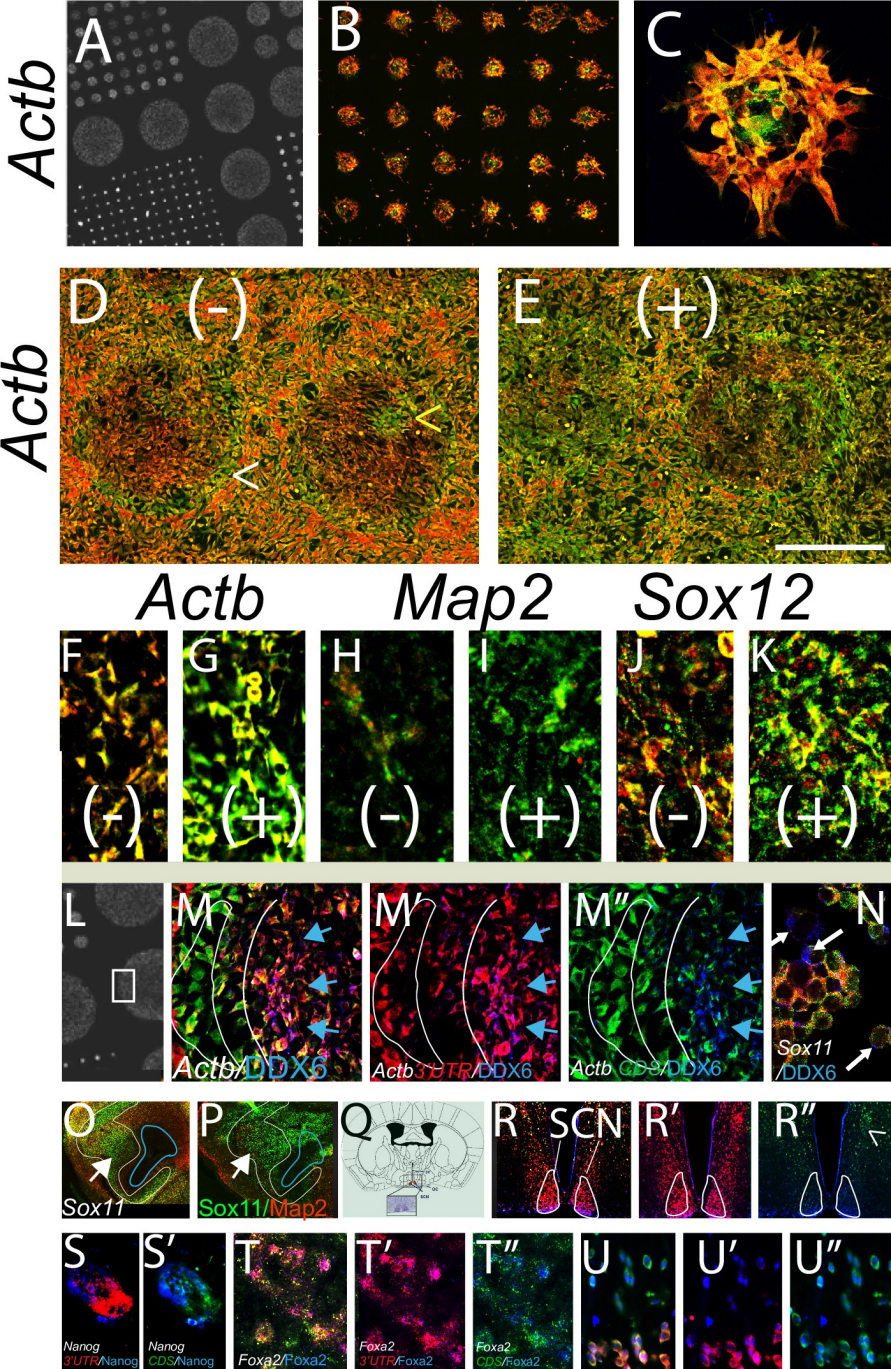
3’UTR/CDS ratios change with cell state. (A) Photo of Cytoo^r^ chip with circles 800 to 80 um arranged across chip. (B,C) In 80 um circles, 3T3 cells consistently show high *Actb* CDS in center cells and high 3’UTR in perimeter cells (technical replicates n >30–50 circles/chip, biological replicates n = 2). In highly confluent cultures cells grow outside the circle. Under normal conditions (D) all cells show very high 3’UTR/CDS except cells at circle border that show high CDS/3’UTR. Stress challenge with 30 min sodium azide (NaZ) (E; + denotes NaZ) causes most cells to express higher *Actb* CDS compared to 3’UTR (D,E are matched photos). Mini-islands of cells that express the opposite ratio from the majority can be seen, suggesting that the 3’UTR/CDS ratio is due to the state of the cell, not to a hybridization artifact of the chip (Fig 4D; yellow carrot). For three different genes, all increase their 3’UTR/CDS ratio after stress, (F,G) *Actb*, (H,I) *Map2*, (J,K) *Sox12* (n = 10 technical replicates, 2 biological replicates). (L-N) Localization of P-body phase marker DDX6 in relation to 3’UTR/CDS. (L) region of Cytoo^r^ chip shown in M-M”. DDX6 is only found in cells with *Actb* 3’UTR (M’; *Actb* 3’UTR red, DDX6 blue), not with cells that express primarily CDS (M”; *Actb* CDS green, DDX6 blue); blue arrows. In Neuro2A cells neither the 3’UTR nor CDS of *Sox11* colocalizes with DDX6 (N; white arrows show DDX6 label in blue). (O,P) Sox11 antibody stain shows same pattern as *Sox11* CDS in similar but non-adjacent section (white arrows and outline; blue outline shows ventricle) (outline from O superimposed onto P). (Q) schematic showing location of suprachiasmatic nucleus SCN (clock center) in the adult brain. (R-R”; *clock* 3’UTR red, CDS green, clock protein, blue). Note that Clock protein shows greater co-localization with the *c lock* 3’UTR (in SCN outlined in white), than with cells with high *Clock* CDS; R” carrot. (S,S’) *Nanog* mRNA and protein in hair bulb (S,S’; *Nanog* 3’UTR red, CDS green, nanog protein blue). Note that in these cells nanog is more highly co-localized with the *Nanog* CDS than 3’UTR. (T-U”) *Foxa2* 3’UTR, red, CDS green, protein blue. (T-T”; embryonic dopamine neurons, U-U”; embryonic dorsal root ganglion neurons). Note that the Foxa2 protein localizes with the *Foxa2* 3’UTR in DA neurons but equally with 3’UTR and CDS in migrating DRG neurons. Scale bar, A; 1700, B; 450, C; 90, D-E; 600, F-K; 150, L; 1200, M,M’, M”; 300, N; 180, O,P; 650 Q; 5700, R, R’, R”; 900 S, S’75, T,T’,T” 3000, U; 430 um.

These data demonstrate that our ISH procedure is specific and sensitive and can distinguish differential signal in closely juxtaposed cells (Fig 1). Further there is an extremely penetrant use of differential cognate 3’UTR to CDS expression in developing and adult, neural and non-neural tissues for many if not all genes examined (Figs 1–4 [2], and unpublished).

### Cellular compartmentalization of the i3’UTRs

As shown previously *in vivo* previously [1, 2], when both the CDS and cognate 3’UTR are expressed in a cell, they may be localized to different cellular compartments (i.e., nuclear vs. cytoplasmic). We examined if this localization is fixed or dynamic, comparing two genes- in clustered versus migrating embryonic peripheral neurons. In clustered neurons, the 3’UTR sequences of two cytoskeletal genes, *Map2* and *Actb* are restricted to the nucleus, but in migrating neurons the *Map2* (but not *Actb*) CDS localizes to the cytoplasm (Fig 1O–1R; R carrots). Thus, 3’UTR and CDS localization is dynamic, varies from gene to gene in a given cell, and is dependent on the cellular state.

We likewise examined compartmentalization within neurons, which are unique due to their extended axonal processes- with many important cellular processes including translation of mRNA taking place in the axons [21–23]. A screening of >20 genes showed four with prominent differential 3’UTR to CDS in or around axons (Fig 1S–1Y). In spinal cord *Nurr1* shows extreme high i3’UTR (with no or low CDS) expression in cells that line specific axons detected with the marker TAG1 (Fig 1W), whereas *Otx2* (with Fig 1X and 1Y) and *Wnt1* (not shown) show high i3’UTR expression within the axons (with no or low CDS) (S1N-S1P Fig in S1 File; see O). Further, the *Marcks* gene shows high 3’UTR expression in the axons of peripheral dorsal root ganglion neurons, whereas the cell bodies of these same neurons produce predominantly *Marcks* CDS (with Fig 1S and 1T). Thus 3’UTRs and their cognate CDS’ may be differentially localized to cell bodies and/or axons.

### Differential 3’UTR to CDS expression in proliferating cells

Due to the strong use of differential 3’UTR to CDS expression in genes involved in developmental processes [2], we examined the 3’UTR to CDS expression of the critical early pluripotent genes (PpGs); *Myc*, *Nanog*, *Sox2*, *Oct4* and *Klf4*. Each PpG was compared to 11 other genes in two systems, the E12 olfactory epithelium (OlfE) (Fig 2A), as well as the developing whisker and hair niche (Figs 2H and 3), which were selected because proliferating progenitors and differentiating progeny have clear and stereotyped spatial locations. In the OlfE subventricular zone (SVZ) all PpGs are expressed and most show striking differential 3’UTR to CDS expression (Fig 2B–2G; S2A-S2H Fig in S1 File). At low power *Sox2* and *Ascl1* (a pro-neural gene used as a control) show unique 3’UTR to CDS patterns across the OlfE with *Sox2* displaying graded differential 3’UTR to CDS expression, and *Ascl1* with more coincident 3UTR/CDS expression-both show identical patterns bilaterally (with Fig 2B and 2C;*). Within the SVZ neural progenitors are located apically and more differentiated cells basally, and radial glia-labeled by Nestin-span the distance. We asked whether PpG 3’UTR to CDS expression ratio correlates with these subregions. All the PgGs except *Myc* show preferential localization of their 3’UTR to apical cells, with isolated CDS throughout the radial glia (labeled by Nestin) into the basal regions (Fig 2E–2G; S2G, S2H Fig in S1 File). For *Myc* both the 3’UTR and CDS are largely restricted to the SVZ apical progenitor region (Fig 2E; yellow carrot, S2E Fig in S1 File).

Because whisker (Fig 2H) and hair follicle niches (Fig 3A–3C) are stereotyped structures where progenitor, proliferative, and other cell domains have been defined anatomically [24, 25] we were able to examine whether 3’UTR to CDS expression correlates with anatomical structure and/or cell proliferation. At postnatal day 2, differential PpG 3’UTR to CDS expression is detected in whisker sub-regions even within early condensates (with Fig 2I and 2J; carrots show developing whiskers). More mature whiskers with clearly visible dermal papillae (DP) show differential 3’UTR to CDS expression for all the PpGs (with Fig 2I and 2J; top two carrots and S2M, S2N Fig in S1 File). *Myc* shows equal 3’UTR to CDS in the proliferative epithelial region (the Shh+ cell region (Gonzales and Fuchs 2017), *Shh* 3’UTR and CDS are both expressed: with Fig 2K and 2M; carrots). In contrast, in the closely apposed but distinct dermal papilla, *Myc* shows much greater 3’UTR compared to CDS expression (Fig 2M; inside green circle). *Sox2* on the other hand, is necessary for DP differentiation [26] and shows equal 3’UTR to CDS expression (Fig 2L; other PpGs in S2I-S2L Fig in S1 File). The juxtaposition of dramatically different 3’UTR to CDS ratios for the PpGs in closely apposed anatomical structures underscores that 3’UTR to CDS ratios are very tightly regulated, showing different or reciprocal relationships in distinct biological structures. Importantly, the 3’UTR to CDS ratio for the PpGs may be critical in developmental events such as neurogenesis and hair formation.

To more closely examine the relationship between differential 3’UTR to CDS ratio and cell proliferation we took advantage of the fact that in the hair follicle the proliferative Ki67+ cells exist below the “critical line of Auber”. Above this line are the non- proliferating “pre-cortex” cells. Based on the strong localization of PpG 3’UTR expression to stem cells in neurons, we hypothesized that PpG’s may show high i3’UTR expression in the Ki67 cells. We found, first, that across serial sections very different patterns of expression are seen for individual genes and each pattern replicates across hair follicles, within a section (Fig 3D–3F; S3A-S3C Fig in S1 File). Examining expression above and below the line of Auber, we found dramatic and abrupt shifts in 3’UTR to CDS expression for *Oct4*, *Klf4* and *Nanog* (as well as *Nurr1*and *DCC*) at this line, with high 3’UTR compared to CDS expression in the Ki67+ cells, shifting to high CDS compared to 3’UTR in Ki67- pre-cortex cells (with Fig 3J and 3L–3N; dotted line is line of Auber). *Myc* showed more coincident expression with somewhat higher 3’UTR than CDS in Ki67+ cells compared to pre-cortex (with Fig 3K and 3K’). In contrast *Sox9* and three other *Sox* genes showed similar 3’UTR to CDS throughout (Fig 3G; S3F, S3G Fig in S1 File). *Shh* and *Ntn1* (used as a control) both showed asymmetric localization, in subsets of the Ki67+ cells (with Fig 3H and 3I; S3D, S3E Fig in S1 File). For each of the 16 genes, expression was independently confirmed to be in hair follicles by scRNAseq data [27] or by Tabula Muris [28].

### Cell state and 3’UTR to CDS ratio

These studies show that there is dynamic differential 3’UTR to CDS use during development. We asked whether we could induce 3’UTR to CDS ratio changes by exposing cells to stress. For this NIH3T3 (3T3) cells were plated on prepatterned glass chips (Cytoo^r^) (Fig 4A; in low confluence cultures the cells attach only to the substrated circles). In the smallest circles (80 um)- we find, dramatically, that cells in the center of each circle show high Actin (*Actb)* CDS compared to 3’UTR, whereas the cells at the perimeter all show high 3’UTR and low CDS; this pattern replicates exactly across more than 30 circles/chip (with Fig 4B and 4C). In confluent cultures where cells grow onto the glass (with Fig 4D and 4E) we examined the *Actb* 3’UTR/CDS ratio in stressed vs non-stressed cultures. In the absence of stress most 3T3 cells express higher *Actb* 3’UTR to CDS, except interestingly the cells on the perimeter of the circles (Fig 4D; white carrot). After stress, most cells switch to express high *Actb* CDS with low 3’UTR (Fig 4E). We also compared the 3’UTR to CDS ratios for three different genes, *Actb Map2* and *Sox12*, and found that for each of these genes there is an increase in their CDS levels compared to their 3’UTR levels after stress (Fig 4F–4K).

### i3’UTR or CDS co-localization with other markers

Of interest of course is how 3’UTR sequences (or CDS sequences) become sequestered within cells. For this we examined the co-localization of various probe sets to specific antibody markers for the nuclear membrane (Lamin), P-body phase regions (DDX2, Rcd2, xrn1), endosomes (Lamp1), and apoptotic cells marked by activated Caspase3. Unlike Ki67 which is highly correlated with the i3’UTR for many genes (Figs 2 and 3), we saw no preferential localization of 3’UTR or CDS for activated Caspase3 or Lamp1 for any gene examined (2–4 for each protein). Because mRNA is often localized to P-bodies we examined DDX6 and other P-body markers. We found that in 3T3 cells, the *Actb* 3’UTR (with or without CDS) was highly colocalized with P-body markers (Fig 4L–4M”; shown is DDX6). For CDS that was expressed with low or no 3’UTR, there was no co-localization (Fig 4M”). While striking for *Actb* in 3T3 cells, neither the *Sox11* 3’UTR nor CDS showed co-localization with DDX6 in 3T3 or Neuro2A cells (Fig 4N; shown is Neuro2A). Thus, while 3’UTR sequences may co-localize with P-bodies (with or without the CDS), this is not a requisite location. We saw similar but less robust colocalization of some 3’UTR sequences with Lamin. Because these localizations appear gene and cell dependent, a more extensive analysis was not pursued here, however analyses in individual biological contexts is likely to be informative.

Equally important is how 3’UTR to CDS ratios correlate with cognate protein levels. We reported previously that for two cytoplasmic proteins, a high 3UTR/CDS ratio correlated with low protein expression [2]. Upon further investigation we found that in the systems studied here Sox2, Sox11 and Nanog proteins are also often likely detected in cells that have high or equal CDS/3’UTR vs those with high 3’UTR to CDS (Figs 2F, 4O, 4P, 4S and 4S’). However, after examining multiple probe sets and cognate proteins; we found some cases, particularly transcription factors, that did not follow that rule, i.e., in those cases we observed preferential localization of the protein with the cognate 3’UTR, this was true for Clock and Foxa2 (with Fig 4R, 4R”, 4T and 4U”), Klf4 and Oct4, and Nanog in some cases (not shown). Interestingly, Foxa2 protein is highly co-localized with the *Foxa2* 3’UTR in embryonic DA neurons in culture but not in same age peripheral neurons (with Fig 4T and 4T” vs 4U and 4U” respectively). While it was unexpected that some proteins would show higher colocalization with cells that are expressing higher cognate 3’UTR (rather than CDS), this was observed across multiple experiments and cell types with a number of different antibodies to different proteins. This also was not an antibody artifact, as not all antibodies showed such co-localization with the 3’UTR sequences). It is striking that in these cases, protein was more highly localized to cells with high expression of the cognate 3’UTR rather than the CDS.

### In silico analysis of i3’UTRs across tissues

**Fig 5:**
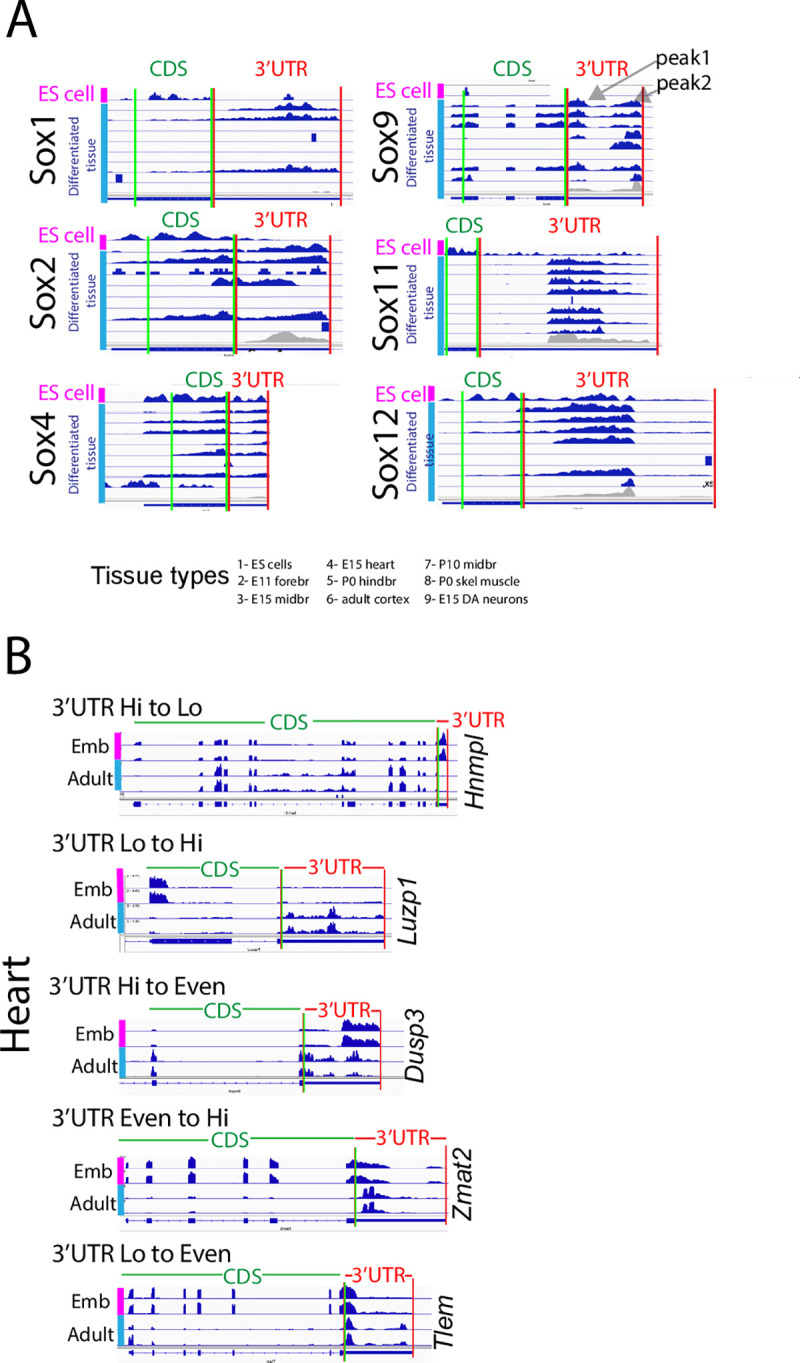
Widespread differential 3’UTR/CDS use is reflected in RNAseq data. (A) RNAseq data from 9 tissues showing 3’UTR and CDS expression for select *Sox* genes. For each gene, red vertical lines denote 3’UTR region, green denote CDS. Top trace for each gene (pink sidebar) is embryonic stem cell (ES) cell. Next eight traces (blue sidebar) show differentiated tissues, the first seven from open source Geo; noted at bottom, and the last, in-house RNAseq from E15 dopamine neurons [2]. As noted in [1, 2] and shown in gray, RNAseq traces are not uniform across the entire 3’UTR and can be divided into “peaks” and “valleys” (e.g., see *Sox9*; peak1 and peak 2; valley in between) (see text for definition). Each *Sox* gene shows greater (or in one case equal) 3’UTR to CDS expression in differentiated tissues. ES cells show the opposite, with high CDS/3’UTR. (B) Representative genes from embryonic (Emb; pink bars) and adult (Adult; blue bars) heart databases (each with two replicates) illustrate 3’UTR/CDS ratio changes over time; e.g., *Hnmpl* 3’UTR/CDS changes from high to low, *Luzp1* from low to high. CDS—3’UTR junction marked by double red-green line and labels above.

**Fig 6:**
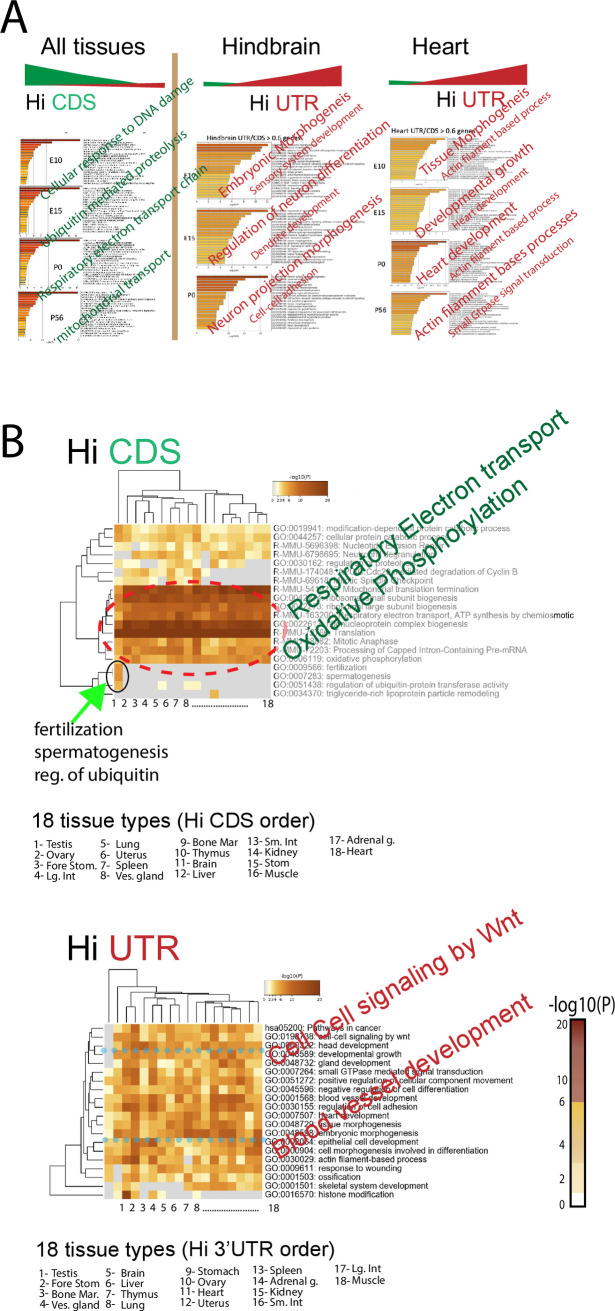
Unbiased analysis shows that Hi 3’UTR/CDS genes define tissue specific properties. (A) Gene Ontology (GO) categories for Hi CDS and Hi 3’UTR (see methods for gene selection criteria) genes for hindbrain and heart data sets. Right columns; top GO for Hi 3’UTR genes, left column; top GO for Hi CDS genes. GO categories are listed in black with examples shown in larger colored diagonal text. (B) Top GO categories across 18 tissues- heat map shows significance for each tissue. Order of tissues for Hi CDS is different from Hi UTR; see legend. In all cases the top Hi CDS GO (dotted red circle) categories are oxidative phosphorylation, electron transport and other basic cellular mechanisms. Hi 3’UTR GO categories vary by tissue, are tissue specific and show no obvious pattern (bottom heat map); blue dotted line shows significance variability across tissues for two representative GO categories. Of note, testis Hi CDS GO categories include fertilization and spermatogenesis in addition to basic cellular mechanisms; green arrow, top heat map. Further, testis Hi UTR GO categories are less aligned with other tissues; bottom heat map, column one, note the gray squares.

The ISH studies reported here and by others strongly suggest that a given gene may utilize different 3’UTR to CDS ratios across tissues and across development, and that any particular gene may change this ratio over time. We examined this in an unbiased manner using open-source RNAseq data. In all bulk (Figs 5 and 6), and single cell data sets (not shown) examined (> 50 data sets including the replicates) we identified genes that show different 3’UTR to CDS ratios in different tissues. For example, we examined expression of multiple *Sox* gene family members in 11 randomly chosen data sets; in general we find higher 3’UTR compared to CDS expression across all differentiated tissues (Fig 5A; teal bar). However, embryonic stem cells (ESC) (Fig 5A; pink bars) show the opposite, with *Sox* members showing higher CDS than 3’UTR expression. We further examined changes in the ratio of 3’UTR to CDS for any given gene in one tissue over time. For this we queried different aged heart tissue (Fig 5B) and found many examples; one is *Hnmpl* which shows high 3’UTR to CDS in the embryo but low 3’UTR to CDS in the adult (Fig 5B; top traces). In contrast, *Dusp3* shifts from high 3’UTR to CDS in the embryo to comparable levels in the adult (Fig 5B; middle traces). Thus, as predicted from ISH studies, genes change their ratio depending on the cell state, and/or maturation level. All heart data sets were generated by the same investigator, arguing against the possibility that differences are due to library generation or sequencing methodologies.

### Classes of genes that show high 3’UTR to CDS

We next examined which particular genes sets, or categories (GO categories) tend to show a high, or low 3’UTR to CDS ratio. We first examined two tissues: hindbrain and heart across multiple ages. Each sample had two biological replicates (with high reproducibility across replicates in genes “called” Hi 3’UTR; 63% to 88% identity) and we asked, for each tissue and age, whether the GO categories corresponding to Hi (defined as >0.6 on scale of 0 (high CDS) to 1 (high 3’UTR) or Lo 3’UTR genes (defined as < 0.4) (see methods for details) are different. We found, first, that, regardless of tissue or age, the top GO category for Hi CDS (Lo 3’UTR) genes is consistently “translation”, followed by “ribonucleoprotein complex biogenesis” (Fig 6A; left). On the other hand, intriguingly, the top GO categories for Hi 3’UTR genes are instead remarkably specific to both the age and function of the target tissue. In hindbrain, the high ranking GO categories are “embryonic morphogenesis”, “neuronal differentiation”, and “synaptogenesis” in E15 to P0 tissue. Likewise, but heart specific, the top high 3’UTR GO categories for heart are “actin filament based processes” “heart development, “actin filament”, and “Gtpase signal transduction” from E15 to P56 (Fig 6A; right columns). The GO categories of Hi 3’UTR genes then are representative of cell and tissue type with high representation of cell fate determination genes. This is true in spite of the fact that both Hi and Lo 3’UTR genes each represent less than 10% of all the genes expressed in that tissue and are not necessarily the most highly expressed genes.

**Fig 7:**
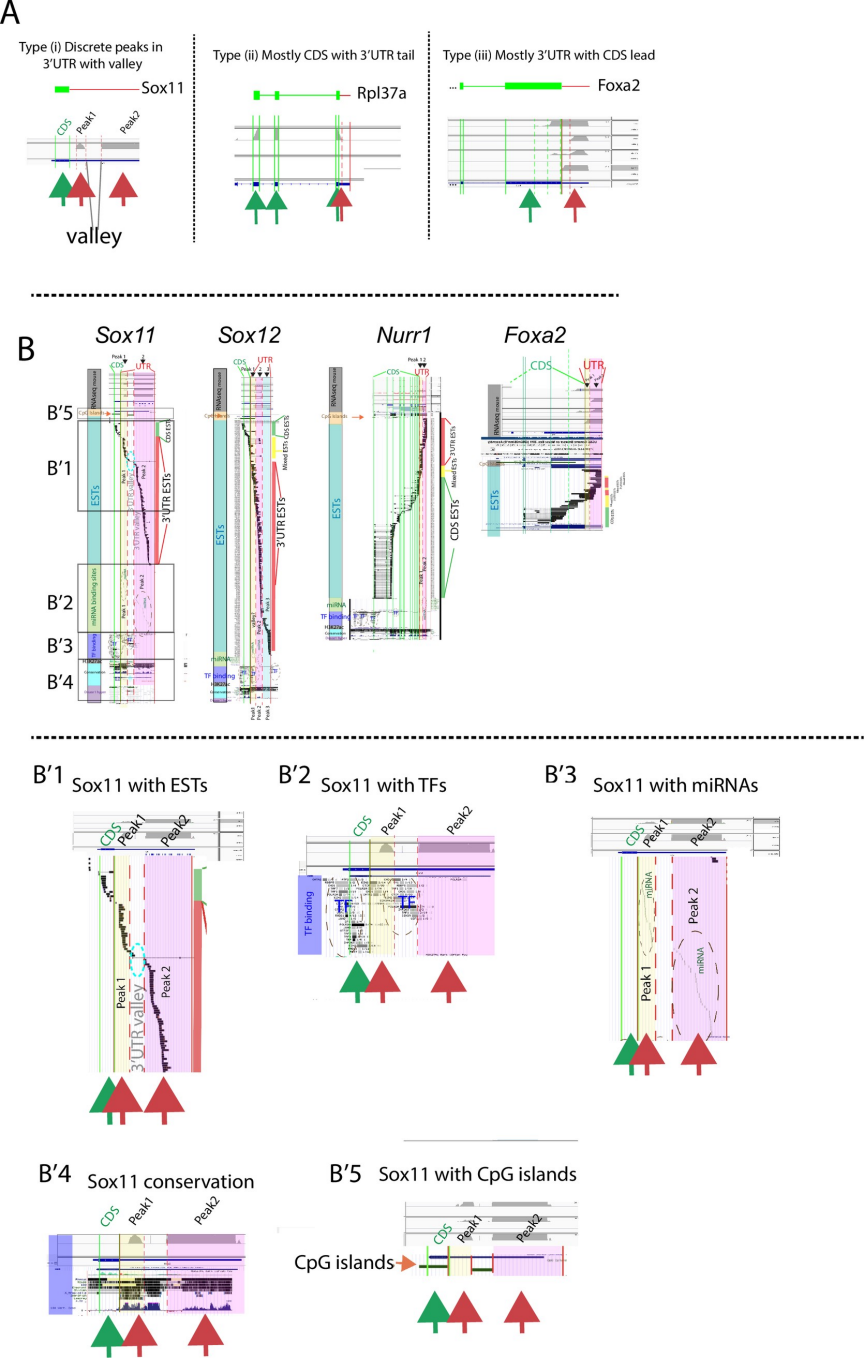
Gene elements and EST sequences correlate with 3’UTR sequence peaks and valleys. (A) illustrative examples of 3’UTR vs CDS peak and valley expression. Gene structure at top. Mouse E15 DA neuron RNAseq trace below. Solid vertical green/red lines denote CDS or 3’UTR; dotted lines denote ends of RNAseq traces. (B) For 4 representative mouse genes; CDS, 3’UTR and prominent RNAseq 3’UTR peaks and valleys are aligned with human gene elements from the UCSC browser http://genome.ucsc.edu/; human Hg19. Human data was used since it contains more complete gene element data. For each gene, color-coded vertical bar denotes elements. Top, gray; E15 midbrain mouse RNAseq traces [2], Orange; CpG islands, Teal; expressed sequence tags (ESTs), Green; miRNA binding sites, Purple; transcription factor (TF) binding sites, Brown; H3K27ac binding sites, Light blue; conservation across species, Lavender; Dnase1 hypersensitive sites (Dnase1 hyper). *Sox11* is first gene on left. Below, (B’1–5) are expanded views of *Sox11* data from regions in boxes. Peaks are defined as continuous regions of >150 bp of sequence with FPKM>100 in 4 replicate data sets, valleys show the opposite. Peaks and valley denoted throughout. Peak1 is overlain in yellow, peak2; pink, peak3; blue, valleys are white. Dark red, green and yellow vertical bars to the right of each gene denote ESTs that are uniquely 3’UTR (red, no CDS), uniquely CDS (green, no 3’UTR) or mixed (ESTs that span junction, yellow). Note that *Sox11* has no mixed 3’UTR ESTs, and of 122 total ESTs only 10% have any CDS sequence. No ESTs span the 3’UTR valley; blue dotted circle (B’1), TF binding sites are situated between peaks (B’2), miRNA binding is exclusively within peaks (B’3), conservation is high in peak, and valley regions (B’4), and H3k27ac and CpG islands are in valleys or CDS region not in the peak regions (B and B’5). *Sox12* shows similar profile to *Sox11* except that Peak1 is not as well defined and shows mixed peak/valley characteristics. *Nurr1* shows many more CDS ESTs than 3’UTR ESTs with miRNAs and high conservation only in peak2. Dotted red lines denote 3’UTR peaks, dotted green lines, CDS peaks. *Foxa2;* aligned with ESTs only, is shown to illustrate that not all genes resemble *Sox11* and *12*. For *Foxa2*, CDS ESTs outnumber 3’UTR ESTs with many mixed. Human gene element data was used as mouse data for various elements was unavailable.

While intriguing, we wanted to see if this result held more generally and in the adult, and therefore examined this across 18 adult tissues. We observed similar results across all 18 tissues, as the top GO categories for Hi CDS genes were “respiratory electron transport” and “oxidative phosphorylation” (Fig 6B; top), whereas high 3’UTR GO categories differ by tissue, with few commonalities between them (Fig 6B; bottom heat map), and with GO categories highly representative of tissue type. For example, the GO category “epithelial cell development” is significant in liver, with “blood vessel” in small intestine and kidney (Fig 6B). While we consistently find that the high CDS genes are similar in all tissues and that the top categories are primarily involved in cellular maintenance, we find that some of the categories, although not the most significant, are more tissue-specific. In testis, for example two significant Hi CDS categories are “fertilization and spermatogenesis” (Fig 6B; green arrow; black circle). Liver also shows significance for “triglyceride-rich lipoprotein particle remodeling”. Thus, apart from a preponderance of Hi CDS use amongst cell maintenance genes, there is also some tissue specific use of Hi CDS. Testis is an outlier for high UTR GO categories as well and does not show any of the same GO categories as other tissues (Fig 7C; bottom; black circle).

### i3’UTR and gene sequence elements

Generation of isolated 3’UTR transcripts may occur by de novo transcription or post-transcriptional processing of mRNA, and may potentially differ for different genes. Here we asked whether the 3’UTR “peaks” show close alignment with gene elements that signal transcriptional activity, examining select candidate genes. First, as seen in Fig 5A (for example *Sox9*; gray arrows) and as previously shown in [1, 2], RNAseq expression within a given 3’UTR region is not necessarily continuous with the CDS, nor continuous along the entire annotated 3’UTR, and instead there are “peaks” of 3’UTR expression (defined in Fig 7 legend). From visual analysis we encountered three main patterns of these peaks, which we term types (i), (ii) and (iii), with any given gene showing high pattern reproducibility across tissues (Fig 7A). Type (i) shows multiple discrete 3’UTR peaks that originate and terminate in the 3’UTR proper, with valleys (absence of peaks) between peaks, Type (ii) involves sequence immediately distal to the end of the CDS. spanning continuously into a short 3’UTR; and Type (iii), a long 3’UTR with a short stretch of leading CDS. Because in many cases a given gene will show very similar patterns of 3’UTR peaks and valleys across tissues and data sets (Figs 5 and 6A; see *Sox11*) we examined gene sequence elements upstream, or aligned with, these peaks and valleys. Using data collected from the UCSC browser (http://genome.ucsc.edu/, human data was used) we aligned the following elements: conservation across species, transcription factor (TF) binding sites, CpG islands, H3K27ac peaks, DNase 1 hypersensitivity sites, and miRNA binding sites- to 3’UTR RNAseq peaks and valleys. Remarkably, for some genes, each of these elements shows tight alignment with discrete 3’UTR peaks and valleys. For example, for *Sox11* (selected since the location of the 3’UTR peaks and valleys are highly consistent across tissues), CpG islands, H3K27ac peaks, and transcription factor (TF) binding clusters are located and confined to the valleys immediately upstream of both peak 1 and 2 within the *Sox11* 3’UTR (with Fig 7B, 7B’2 and 7B’5). In contrast, miRNA binding sites (at which miRNAs are expected to bind to 3’UTR sequences) show the opposite-they are completely confined to the peak1 and peak2 regions, never within valleys (Fig 7B’3), consistent with the peaks and not the valleys being the regions of expression. Further, there is extremely high sequence conservation across species within *Sox11* 3’UTR peak 1 and 2, in fact conservation across these peaks is greater than across the *Sox11* CDS (Fig 7B’4). This high degree of evolutionary conservation in 3’UTR sequence is true for some, but not all genes (S4 Fig in S1 File).

We next assumed that i3’UTRs, as well as their particular peaks and valleys should be reflected in EST data, which include 74 million stretches of RNA sequence generated from cDNA libraries and provide a sampling of the naturally-occurring mRNA species. Of the 122 ESTs assigned to *Sox11*, 89% correspond to 3’UTR alone and contain no CDS sequence, with the rest (11%) corresponding to the CDS alone (with Fig 7B and 7B’1 red arrows, 3’UTR, green arrow, CDS). Strikingly, not one *Sox11* EST spans the 3’UTR-CDS junction even though 4% correspond to the 250 bp immediately upstream. Downstream after an EST gap for 750 bp there is an abrupt uptick with 9% of the ESTs corresponding to next 250 bp. As with the CDS to 3’UTR junction, there is not one EST that spans the 3’UTR valley between peak1 and 2 (Fig 7 B’1; blue dotted circle). *Sox12* shows similar patterns, 84% of the Sox12 ESTs correspond to 3’UTR only, 5% to CDS only, and 10% span the junction (Fig 7B). However, for other genes, for example *Nurr1*, most ESTs are uniquely CDS (Fig 7B; green sidebar vs red sidebar). For yet others, in particular those with a Type (iii) pattern (a long 3’UTR with leading truncated CDS peak) there is good EST representation across the CDS-3’UTR junction; an example is *Foxa2*, which shows a high percentage of ESTs that cross the 3’UTR to CDS junction (Fig 7B; right; yellow bar).

In summary for some genes, we find very high 3’UTR conservation across species with gene sequence elements that are perfectly aligned perfectly with observed RNAseq peaks and valleys. For other genes, even though we observe highly differential 3’UTR to CDS expression by ISH (e.g., *Foxa2*), we do not find strict gene element and peak alignment. It may be for some genes, differential 3’UTR to CDS expression is not regulated from within the genome by gene elements, but is post-transcriptional.

## Discussion

We show here that in cells in all tissues, at all ages, and for many if not all genes there is differential expression of coding regions (CDS) and parts or all of their 3’UTR sequences. This occurs in a non-random fashion, and within a tissue, gene ontology analysis shows that genes displaying high 3’UTR to CDS expression are likely to be involved in the particular functions of that tissue, whereas genes with more equal CDS and 3’UTR expression, or higher CDS than 3’UTR expression, are likely involved in more general cell maintenance functions. In addition, 3’UTR to CDS ratios are not fixed but are dynamic and change as a cell changes state or developmental age. Within a 3’UTR we (and others) [1, 2], find non-continuous expression with peaks and valleys of RNA that is expressed-and with strikingly similar peaks and valley expression for many genes across tissues. At least for some genes, the evolutionary conservation of these peaks suggests the importance and biological relevance of these isolated, expressed, 3’UTR sequences. Further, for some genes, the peaks and valleys are aligned with miRNA and TF binding, as well as H3K27ac marks, suggesting that there may be elements intrinsic to the gene sequences that mediate de novo formation and expression of isolated 3’UTRs. H3k27ac is known to be involved in increased expression of lncRNAs [29, 30].

### Differential 3’UTR to CDS usage by ISH and cellular localization assessed by ISH

The use of multiple controls shows our ISH procedure is robust and sensitive, and the i3’UTR expression patterns allow interesting conjectures. By analogy to the known functions of lncRNAs, (which, by definition, include i3’UTRs), isolated 3’UTRs may bind to specific miRNAs and RNA binding proteins (RBPs) [31, 32], and are likely able to add positive or negative value to the cognate protein’s expression and/or ability to function. One interesting example is Nurr1, whereas studies of Nurr1 protein show restricted expression, we find widespread *Nurr1* 3’UTR expression in both developing and adult tissues, often in the absence of high or even detectable levels of CDS. Genes that are thought to have very restricted expression based on their protein or CDS location may have widespread and high levels of 3’UTR expression in other cells. Nurr1 is one of three orphan steroid receptors sub-family members that are all suggested to be tumor suppressors [33–35], however their role can vary depending on cellular context [33]. It is interesting to speculate that an interplay between isolated *Nurr1* 3’UTR levels and Nurr1 protein add to this contextualization. Here we showed high levels of *Nurr1* isolated 3’UTR sequences in proliferating cells (in the Ki67+ cells of the deep hair follicle) and high levels of *Nurr1* CDS in just adjacent Ki67- cells. Nurr1 is known to affect cell cycle [36], and it may be that highly expressed 3’UTR sequences in proliferating cells function in cell cycle dynamics, either together with their cognate protein or other proteins, or in the absence of their cognate protein.

Importantly we found many instances of isolated 3’UTR expression in the nucleus. Further, while proteins are often localized to cells with equal levels of cognate 3’UTR and CDS, or higher levels of their cognate CDS, we find multiple notable exceptions: certain transcription factors are found to preferentially colocalize with their cognate i3’UTR sequences in the nucleus in some cells and some instances. While we cannot verify that the CDS is entirely absent from these regions, what is clear is that there is less CDS than in nearby cells, so we can conclude in such instances that there is preferential localization of protein with i3’UTR sequences compared to CDS sequences. It is interesting to speculate that 3’UTR sequences in the nucleus may interact with their cognate protein in some way, e.g., to stabilize/and or sequester it to delay degradation or temporarily prevent function. Further studies are required to examine this. The specific localization of high levels of 3’UTR sequences to either the nucleus, or to axons, and the dynamic nature of these localizations have significant implications not only for nervous system axonal guidance synaptogenesis and plasticity, but for other cellular functions as well.

### Elusive gene families

Pinning down the precise or even general role of large gene families such as the *Sox* genes has been elusive due to imprecise knowledge about their gene and protein expression, as well as partial biological redundancy as evidenced by only small phenotypes after gene deletion in some cases. Knowledge and manipulation of the distinct 3’UTR to CDS expression patterns of members of these gene families provides an additional handle with which to examine their function in neural and other development processes. Dual ISH of *Sox* mRNA expression reveals intricate patterns for each *Sox* gene in the nervous system with precise and abrupt changes in the use of the 3’UTR and/or the CDS during neuronal development. Extremely intriguing *Sox* gene 3’UTR to CDS patterns were observed in other tissues as well, and require more detailed exploration.

In addition, the Netrin ligand-receptor family contains multiple distinct receptors, including four members of the Unc5 family, which have roles in neuronal and vascular development as well as tumor suppression [37, 38]. The very robust expression of *Unc5d* 3’UTR in blood vessels suggests that perhaps this alternate family member with highly expressed 3’UTR sequences may in some way enhance or modulate the specific known interactions of Netrin-1 and Unc5B in this tissue [39, 40].

### Stem cell biology

In two different stem cell niches we observed highly differential 3’UTR to CDS expression of the core pluripotent gene (PpG) network in progenitor cells. Importantly, in adjacent sections, non-PpG genes; (i.e., *Shh* and *Ascl1)* show more restricted patterns, and with more coincident 3’UTR to CDS expression. This is notable for two reasons, first PpG gene expression has not been highly documented outside of pluripotent stem cells. In agreement with other studies, we find that PpG protein is low in these systems (not shown), but their mRNA levels are robust, and protein is reliably detected in a reasonable cohort of cells expressing the cognate mRNA. We expect that the precise regulation of the 3’UTR to CDS ratio, and thereby regulation of PpG protein expression might be critical for stem/progenitor cell function, possibly affecting the onset, duration and magnitude of proliferation and quiescence. It may be that PpG protein is used sparingly, but that cells maintain specific 3’UTR to CDS expression levels, possibly to provide precise and titrated levels of protein. Secondly, we find abrupt changes in 3’UTR to CDS ratios in closely apposed but morphologically distinct tissues, such as *Sox2* and *Myc* in the whisker dermal papillae and a number of genes in Ki67+ vs Ki67– cells in the hair follicle. Sox2 is critical in DP development [26, 41] and shows equal 3’UTR to CDS in DP cells, whereas *Myc*, known for its role in proliferation, shows high 3’UTR. Interestingly, DP cells serve as a reservoir of stem cells (IPSC cells derive from the DP [42–44]; the high *Myc* 3’UTR- low CDS in these cells may allow them to maintain a non-proliferative, quiescent stem cell state.

### Dynamic 3’UTR to CDS

In addition to multiple genes that show specific 3’UTR and CDS expression, these ratios are dynamic and depend on the cell state- we show they change across development [2], after stress, or with proliferative challenge.

While some genes showed prominent localization of their 3’UTR sequences to P-bodies, marked by DDX6, or the nuclear membrane marked by Lamin, this was not consistent across genes and or cell types and does not appear to be requisite. However, we never observed isolated CDS co-localized with these markers. Examination of individual i3’UTR sequences with these structures in particular contexts will be informative.

### Bioinformatics

This bioinformatic study is the first to systematically examine gene classes that are likely to express high 3UTR/CDS ratios or the reverse, high CDS compared to 3’UTR. They confirm and extend previous findings from our work and others using RNAseq and ISH [1–3]. Here, using random select open source RNAseq data sets we find that differential 3’UTR to CDS expression is commonly observed, and appears highly regulated, both in the expression of either the 3’UTR or CDS and in the use of particular 3’UTR regions. We further show that, although isolated 3’UTR sequences are not always continuous with the CDS, nor are they continuous along the length of the 3’UTR, their “peaks” and “valleys”- at least for some genes such as *Sox11* and *12* align perfectly with various gene elements. For both genes the 3’UTRs are highly conserved across species, with the peaks preceded by TF clusters, DNase1 hypersensitive sites, CpG islands and H3K27ac marks, all hallmarks of gene transcription initiation. Further, and in contrast, known miRNA binding sites align perfectly with the RNAseq peaks and not with the valleys. Finally, the library of known ESTs aligns exactly with the peaks and not with the valleys. Both *Sox11* and *12* have few exons and long 3’UTRs. For other genes, even though we see highly differential 3’UTR to CDS expression by in situ hybridization and RNAseq, these elements may be, but are not necessarily as perfectly aligned. It is possible that for some genes, the expression of isolated 3’UTR sequences is genome driven, while for others post-translational mechanisms are important. Further study, both bioinformatic and by mechanistic dissection, will shed light on these possibilities. Again, because it appears to be gene dependent, we provide examples of “types” of genes observed.

Finally, by examining the GO categories of high CDS and high 3’UTR genes across multiple tissues we find that high 3’UTR genes are tissue specific- related to the particular functions of, or the developmental state of each tissue. In contrast the bulk of significant GO categories for high CDS genes are related to cell maintenance. However, in testis there were also significantly expressed GO categories for high CDS genes that were tissue specific. A possible interpretation of those data is that cells may use high isolated 3’UTR expression for tissue specific genes when the cell is in a metastable state, either developmentally or when carrying out its specific function (i.e., Gtpase signaling in adult heart). Conversely, the identification of i3’UTR genes may inform us about a tissue at hand, either in chronic (cancer) or acute (viral infection) disease states.

In sum, we have shown the widespread and non-random use of differential 3’UTR to cognate CDS expression across tissues, development and cell state. This data shows there is a strong proclivity for high 3’UTR to CDS in cells and for genes involved in transition states, during both proliferative and developmental maturation events.

## Materials and methods

Animal procedures were approved by the Stanford University Institutional Care and Use Committee and followed the National Institutes of Health guidelines for care and use of laboratory animals. All work was given written approval by IACUC protocol APLAC-32091, with expiration date 11/18/22. For this 1–2 animals were sacrificed/month in order to harvest tissue for in situ hybridization and immunohistochemical analyses. Decapitation was only used for embryos harvested from recently euthanized pregnant female. Pregnant females were sacrificed by CO2 inhalation followed by cervical dislocation, with embryos dissected from the carcass on ice and decapitated before dissection and harvesting embryonic tissue for histological analysis. For tissue harvested from animals older than E18, animals were anesthetized at various postnatal ages by ketamine/xylazine IP injection, and sacrificed via transcardial perfusion of paraformaldehyde.

### Fluorescence in situ hybridization (ISH) and immunochemistry

Two color fluorescence ISH was performed as previously described [2], using the TSA Plus Cyanine 3 & Fluorescein (NEL753001KT, PerkinElmer, Waltham, MA) according to the manufacturer’s instructions. Briefly, embryonic and adult tissues, or cells in culture were fixed with 4% paraformaldehyde (PFA) in 0.1 M phosphate buffer (PB). Tissues were cryoprotected, embedded in OCT and stored at -80C until 16 μm cryostat sections were collected (Lecia, Buffalo Grove, IL) onto Superfrost plus glass slides (Invitrogen). For both tissues and cells, after post-fixation in 4% PFA/PB, Proteinase K treatment, acetylation (1% triethanolamine and 0.25% acetic anhydride and prehybridization (50% formamide, 5X SSC, 5X Denhardt’s solution, 0.5 mg/ml Herring sperm DNA and 250 μg/ml Yeast tRNA) at room temperature for 1 hour, tissue sections were hybridized overnight (cells for 3 hours) with fluorescein labeled CDS and Digoxygenin (DIG) labeled probes 3’UTR probes at 56°C. Specimens were then sequentially post-stained with fluorescein or Cy3 chromogens, respectively, and the corresponding Alexa Fluor 647 secondary antibodies and Hoechst 33342 for DNA staining, and mounted with Mounting Medium. Antibodies in S1 Table in S1 File. In situ probes in S2 Table in S1 File.

#### Cell culture

NIH3T3 cells were grown at 37^’^C in DMEM, 10% FBS and 0.5 mg/ml Penicillin Streptomycin Glutamine. Sodium azide (Sigma/Aldrich) was added to cell media 30 min before fixation.

#### Micropattern cell culture

Micropatten CYTOO (Arena A, CYTOO, France) chip cell culture was performed as previously described (Morgani S. et. al.). Patterns of 800, 500, 225, 120 and 80 um circles are dispersed over the full surface, with 25, 144, 576, 900 and 1296 colonies of each size respectively. Briefly, 700 ul drops of 20 mg/ml Laminin (L20202)(Sigma) in PBS without calcium and magnesium (PBS-/-) in were spotted onto parafilm lain in a 15 cm tissue culture plate. After one wash in PBS-/-, chips were inverted on top of the drops for 2h at 37°C. They were then washed 5x with PBS-/- and a single cell suspension of 3T3 cells (2 x 10^6^) was evenly plated onto chips lain in 6-well plates for two days in complete DMEM medium before fixation and ISH.

### Bioinformatic analyses

#### Data processing of the raw sequence reads

Raw data was downloaded from the ENCODE or GEO databases and quality control (QC) was carried out for all FASTQ files using the FastQC tool (http://www.bioinformatics.babraham.ac.uk/projects/fastqc/). All raw reads were aligned to mm10 reference genome using STAR (v2.7.3a) with default settings and aligned files were filtered with minimum mapping quality 20 using samtools (v1.11). Alignment files (BAM) from ENCODE were downloaded directly. To obtain expression matrices for 3’UTR and CDS sequences, we quantified gene coverage for 3’UTR and CDS respectively using the bedtools (v2.17.0) function coveragebed, with the alignment file (BAM). From this output, we were able to obtain gene effective length per gene as the number of bases with non-zero coverage, and read count /per gene. CDS or 3’UTR expression was normalized by their library size as reads per million total mapped reads and quantified as transcripts per base pair using the following formula: Genes with > = 5 mapped reads are considered expressed.

CDS or 3’UTR expression = mapped reads assigned to each gene’s CDS or 3’UTR/(gene effective length (kb) x total mapped reads (millions)).

For each gene, the relative abundance of 3’UTR to CDS is quantified and expressed as the fractional ratio of (3’UTR mean coverage)/ (3’UTR mean coverage + CDS mean coverage) which ranges from 0, (highest ratio of CDS to 3’UTR) to 1, (highest ratio of 3’UTR to CDS). Gene ontology (GO) enrichment analysis was performed using the Metascape online tool (http://metascape.org/gp/index.html) [45] and the top GO categories were selected according to the binomial P values.

## Supporting information

S1 File (ZIP)

## References

[1] Mercer TR, et al. (2011) Expression of distinct RNAs from 3’ untranslated regions. *Nucleic Acids Res* 39(6):2393–2403. 10.1093/nar/gkq1158 21075793 PMC3064787

[2] Kocabas A, Duarte T, Kumar S, & Hynes MA (2015) Widespread Differential Expression of Coding Region and 3’ UTR Sequences in Neurons and Other Tissues. *Neuron* 88(6):1149–1156. 10.1016/j.neuron.2015.10.048 26687222

[3] Malka Y, et al. (2017) Post-transcriptional 3 -UTR cleavage of mRNA transcripts generates thousands of stable uncapped autonomous RNA fragments. *Nat Commun* 8(1):2029. 10.1038/s41467-017-02099-7 29229900 PMC5725528

[4] Florczyk M, Brzuzan P, Krom J, Wozny M, & Lakomiak A (2016) miR-122-5p as a plasma biomarker of liver injury in fish exposed to microcystin-LR. *J Fish Dis* 39(6):741–751. 10.1111/jfd.12406 26345281

[5] Mayr C (2017) Regulation by 3’-Untranslated Regions. *Annu Rev Genet* 51:171–194. 10.1146/annurev-genet-120116-024704 28853924

[6] Barrett LW, Fletcher S, & Wilton SD (2012) Regulation of eukaryotic gene expression by the untranslated gene regions and other non-coding elements. *Cell Mol Life Sci* 69(21):3613–3634. 10.1007/s00018-012-0990-9 22538991 PMC3474909

[7] Hesketh J (2004) 3’-Untranslated regions are important in mRNA localization and translation: lessons from selenium and metallothionein. *Biochem Soc Trans* 32(Pt 6):990–993. 10.1042/BST0320990 15506944

[8] Rastinejad F, Conboy MJ, Rando TA, & Blau HM (1993) Tumor suppression by RNA from the 3’ untranslated region of alpha-tropomyosin. *Cell* 75(6):1107–1117. 10.1016/0092-8674(93)90320-p 7505203

[9] Spicher A, et al. (1998) Highly conserved RNA sequences that are sensors of environmental stress. *Mol Cell Biol* 18(12):7371–7382. 10.1128/mcb.18.12.7371 9819424 PMC109319

[10] Manjeshwar S, Branam DE, Lerner MR, Brackett DJ, & Jupe ER (2003) Tumor suppression by the prohibitin gene 3’untranslated region RNA in human breast cancer. *Cancer Res* 63(17):5251–5256. 14500355

[11] Crerar H, et al. (2019) Regulation of NGF Signaling by an Axonal Untranslated mRNA. *Neuron* 102(3):553–563 e558. 10.1016/j.neuron.2019.02.011 30853298 PMC6509357

[12] Qureshi IA, Mattick JS, & Mehler MF (2010) Long non-coding RNAs in nervous system function and disease. *Brain Res* 1338:20–35. 10.1016/j.brainres.2010.03.110 20380817 PMC2883659

[13] Gibb EA, Brown CJ, & Lam WL (2011) The functional role of long non-coding RNA in human carcinomas. *Mol Cancer* 10:38. 10.1186/1476-4598-10-38 21489289 PMC3098824

[14] Rinn JL & Chang HY (2012) Genome regulation by long noncoding RNAs. *Annu Rev Biochem* 81:145–166. 10.1146/annurev-biochem-051410-092902 22663078 PMC3858397

[15] Mercer TR & Mattick JS (2013) Structure and function of long noncoding RNAs in epigenetic regulation. *Nat Struct Mol Biol* 20(3):300–307. 10.1038/nsmb.2480 23463315

[16] Ng SY & Stanton LW (2013) Long non-coding RNAs in stem cell pluripotency. *Wiley Interdiscip Rev RNA* 4(1):121–128. 10.1002/wrna.1146 23139157

[17] Taft RJ, Pang KC, Mercer TR, Dinger M, & Mattick JS (2010) Non-coding RNAs: regulators of disease. *J Pathol* 220(2):126–139. 10.1002/path.2638 19882673

[18] Perez DS, et al. (2008) Long, abundantly expressed non-coding transcripts are altered in cancer. *Hum Mol Genet* 17(5):642–655. 10.1093/hmg/ddm336 18006640

[19] Hoser M, et al. (2008) Sox12 deletion in the mouse reveals nonreciprocal redundancy with the related Sox4 and Sox11 transcription factors. *Mol Cell Biol* 28(15):4675–4687. 10.1128/MCB.00338-08 18505825 PMC2493363

[20] Wang Y, Lin L, Lai H, Parada LF, & Lei L (2013) Transcription factor Sox11 is essential for both embryonic and adult neurogenesis. *Dev Dyn* 242(6):638–653. 10.1002/dvdy.23962 23483698

[21] Kim E & Jung H (2020) Local mRNA translation in long-term maintenance of axon health and function. *Curr Opin Neurobiol* 63:15–22. 10.1016/j.conb.2020.01.006 32087477

[22] Cioni JM, Koppers M, & Holt CE (2018) Molecular control of local translation in axon development and maintenance. *Curr Opin Neurobiol* 51:86–94. 10.1016/j.conb.2018.02.025 29549711

[23] Preitner N & Flanagan JG (2012) Axonal mRNA translation: an unexpected link to axon survival and the mitochondrion. *Neuron* 73(4):629–631. 10.1016/j.neuron.2012.02.005 22365539 PMC3712866

[24] Fuchs E, Tumbar T, & Guasch G (2004) Socializing with the neighbors: stem cells and their niche. *Cell* 116(6):769–778. 10.1016/s0092-8674(04)00255-7 15035980

[25] Rompolas P & Greco V (2014) Stem cell dynamics in the hair follicle niche. *Semin Cell Dev Biol* 25–26:34–42. 10.1016/j.semcdb.2013.12.005 24361866 PMC3988239

[26] Driskell RR, Giangreco A, Jensen KB, Mulder KW, & Watt FM (2009) Sox2-positive dermal papilla cells specify hair follicle type in mammalian epidermis. *Development (Cambridge*, *England)* 136(16):2815–2823. 10.1242/dev.038620 19605494 PMC2730408

[27] Joost S, et al. (2018) Single-Cell Transcriptomics of Traced Epidermal and Hair Follicle Stem Cells Reveals Rapid Adaptations during Wound Healing. *Cell reports* 25(3):585–597 e587. 10.1016/j.celrep.2018.09.059 30332640

[28] Tabula Muris C, et al. (2018) Single-cell transcriptomics of 20 mouse organs creates a Tabula Muris. *Nature* 562(7727):367–372. 10.1038/s41586-018-0590-4 30283141 PMC6642641

[29] Liu D, et al. (2020) H3K27 acetylation-induced lncRNA EIF3J-AS1 improved proliferation and impeded apoptosis of colorectal cancer through miR-3163/YAP1 axis. *J Cell Biochem* 121(2):1923–1933. 10.1002/jcb.29427 31709617

[30] Chen F, et al. (2019) lncRNA PLAC2 activated by H3K27 acetylation promotes cell proliferation and invasion via the activation of Wnt/betacatenin pathway in oral squamous cell carcinoma. *Int J Oncol* 54(4):1183–1194. 10.3892/ijo.2019.4707 30720068 PMC6411352

[31] Ulitsky I & Bartel DP (2013) lincRNAs: genomics, evolution, and mechanisms. *Cell* 154(1):26–46. 10.1016/j.cell.2013.06.020 23827673 PMC3924787

[32] Kopp F & Mendell JT (2018) Functional Classification and Experimental Dissection of Long Noncoding RNAs. *Cell* 172(3):393–407. 10.1016/j.cell.2018.01.011 29373828 PMC5978744

[33] Beard JA, Tenga A, & Chen T (2015) The interplay of NR4A receptors and the oncogene-tumor suppressor networks in cancer. *Cell Signal* 27(2):257–266. 10.1016/j.cellsig.2014.11.009 25446259 PMC4276441

[34] Deutsch AJ, Angerer H, Fuchs TE, & Neumeister P (2012) The nuclear orphan receptors NR4A as therapeutic target in cancer therapy. *Anticancer Agents Med Chem* 12(9):1001–1014. 10.2174/187152012803529619 22583411

[35] Deutsch AJA, et al. (2017) NR4A3 Suppresses Lymphomagenesis through Induction of Proapoptotic Genes. *Cancer Res* 77(9):2375–2386. 10.1158/0008-5472.CAN-16-2320 28249906

[36] Sirin O, Lukov GL, Mao R, Conneely OM, & Goodell MA (2010) The orphan nuclear receptor Nurr1 restricts the proliferation of haematopoietic stem cells. *Nat Cell Biol* 12(12):1213–1219. 10.1038/ncb2125 21076412 PMC3059561

[37] Dong D, et al. (2019) UNC5D, suppressed by promoter hypermethylation, inhibits cell metastasis by activating death-associated protein kinase 1 in prostate cancer. *Cancer Sci* 110(4):1244–1255. 10.1111/cas.13935 30632669 PMC6447834

[38] Grandin M, et al. (2016) Structural Decoding of the Netrin-1/UNC5 Interaction and its Therapeutical Implications in Cancers. *Cancer Cell* 29(2):173–185. 10.1016/j.ccell.2016.01.001 26859457

[39] Yang Y, et al. (2007) Axon guidance cue Netrin-1 has dual function in angiogenesis. *Cancer Biol Ther* 6(5):743–748. 10.4161/cbt.6.5.3976 17387275

[40] Prieto CP, et al. (2017) Netrin-1 acts as a non-canonical angiogenic factor produced by human Wharton’s jelly mesenchymal stem cells (WJ-MSC). *Stem Cell Res Ther* 8(1):43. 10.1186/s13287-017-0494-5 28241866 PMC5330133

[41] Salz L & Driskell RR (2017) The Sox2: GFP+/- knock-in mouse model does not faithfully recapitulate Sox2 expression in skin. *Exp Dermatol* 26(11):1146–1148. 10.1111/exd.13396 28636810

[42] Toma JG, et al. (2001) Isolation of multipotent adult stem cells from the dermis of mammalian skin. *Nat Cell Biol* 3(9):778–784. 10.1038/ncb0901-778 11533656

[43] Fernandes KJ, et al. (2004) A dermal niche for multipotent adult skin-derived precursor cells. *Nat Cell Biol* 6(11):1082–1093. 10.1038/ncb1181 15517002

[44] Lavoie JF, et al. (2009) Skin-derived precursors differentiate into skeletogenic cell types and contribute to bone repair. *Stem Cells Dev* 18(6):893–906. 10.1089/scd.2008.0260 18834279

[45] Zhou Y, et al. (2019) Metascape provides a biologist-oriented resource for the analysis of systems-level datasets. *Nat*. *Comm*. 10 (1): 1523–1528. 10.1038/s41467-019-09234-6 30944313 PMC6447622

