# Supplementary material for: Distinct expression of select and transcriptome-wide isolated 3’UTRs suggests critical roles in development and transition states
Source: PLoS One. 2021 May 5;16(5):e0250669. doi: 10.1371/journal.pone.0250669 (PMC8099112; doi:10.1371/journal.pone.0250669)
Supplement: S1 File — (ZIP) [file pone.0250669.s001.zip › Ji et al Hynes Supp Table 2.pdf]

probe name	NCBI mRNA	CDS sequences	CDS size (bp) and l
Sox9	https://www	AGGAAGCTGGCAGACCAGTACCCGCATCTGCACAA<br>CGCGGAGCTCAGCAAGACTCTGGGCAAGCTCTGGAGG<br>CTGCTGAACGAGAGCGAGAAGAGACCCTTCGTG<br>GAGGAGGCGGAGCGGCTGCGCGTGCAGCACAAGAAA<br>GACCACCCCGATTACAAGTACCAGCCCCGGCGGA<br>GGAAGTCGGTGAAGAACGGACAAGCGGAGGCCGAAG<br>CTTCATGGTGTGGTCC	407(735-1142)
Sox11	https://www	AAGATCGAGCGCAGGAAGATCATGGAGCAGTCGCCCG<br>ACATGCACAACGCCGAGATCTCCAAGAGGCTGG<br>GCAAGCGCTGGAAGATGCTGAAGGACAGCGAGAAGA<br>TCCCGTTCATCAGGGAGGCGGAGCGCCTGCGCCT<br>CAAGCACATGGCTGATTATCCCGACTACAAGTACCGGC<br>CGCGCAAAAAGCCCAAGACGGACCCAGCGGCC<br>AAGCCCAGCGCGGGCCAGAGCCCCGACAAGAGCGCGG<br>CGGGCGCCAAGGCAGCCAAGGGCCCCGGCAAGA<br>AGTGCGCCAAGCTCAAGGCGCCTGCGGGCAAGGCGGG<br>CGCGGGCAAGGCGGCGCAGCCGGGGGACTGCGC	428(475-902)
Marcks	https://www	AGGAGAATGGCCACGTAAAAGTGAACGGGGACGCGT<br>CTCCCGCCGCCGCCGAGCCGGGCGCCAAGGAGG<br>AGCTGCAAGCCAACGGCAGCGCCCCGGCCGCCGACAA<br>GGAGGAGCCCGCGAGCGGCAGTGCCGCGACCCC<br>CGCCGCGGCCGAAAAGGATGAGGCTGCCGCGGCCACC<br>GAGCCGGGCGCCGGCGCGGCCGACAAGGAGGCT<br>GCGGAGGCCGAGCCCGCCGAGCCCAGCTCCCCGGCCGC<br>CGAGGCCGAGGGCGCGTCCGCCTCCTCCACGT<br>CGTCGCCCAAGGCGGAGGACGGGGCCGCGCCGTCGCC<br>CAGCAGCGAGACCCCGAAAAAAAAAAAGAAGCG<br>CTTTTCCTTCAAGAAGTCCTTCAAGCTGAGCGGCTTCTC<br>CTTCAAGAAGAGCAAGAAGGAGTCGG	414(492-906)
Sox4	https://www	GCCCAAGAAGAGCTGTGGCCCCAAGGTGGCGGGCAGC<br>TCGGTCGGC<br>AAGCCCCACGCTAAGCTGGTCCCGGCGGGCGGCAGCA<br>AGGCGGCTGCATCGTTCTCTCCAGAGCAAGCTG<br>CCCTGCTGCCCCTGGGGGAGCCCACGGCCGTCTACAAG<br>GTGCGGACTCCCAGTGCGGCCACTCCGGCCGC<br>CTCCTCCTCGCCGTCCAGTGCGCTGGCCACCCCAGCCAA<br>ACACCCTGCCGACAAGAAAGTGAAGCGCGTC<br>TACCTGTTTGGAAGCCTGGGCGCTTCGGCGTCTCCCGT<br>CGGGGGCCTGGGAGCGAGCGCCGACCCCAGTG<br>ATCCACTGGGGTTGTACGAAGATGGAGGC	355(1215-1570)

Nurr1	https://www	GGACGATCCGGGCTCCCTTCACAACTTCCACCAGAACT<br>ACGTGGCCACTACGCATATGATCGAGCAGAGGAAGAC<br>ACCTGTCTCCCGCCTGTCACTCTTCTCCTTTAAGCAGTC<br>GCCCCCGGGCACTCCTGTGTCTAGCTGCCAGATGCGCT<br>TCGACGGGCCTCTGCACGTCCCCATGAACCCGGAGCCC<br>GCGGGCAGCCACCACGTAGTGGATGGGCAGACCTTCG<br>CCGTGCCCAACCCCATTCGCAAGCCGGCATCCATGGGC<br>TTCCCGGGCCTGCAGATCGGCCACGCATCGCAGTTGCT<br>TGACACGCAGGTGCCCTCGCCGCCGTCCCGGGGCTCTC<br>CCTCCAATGAGGGTCTGTGCGCTGTTTGCGGTGACAAC<br>GCGGCCTGTCAGCACTACGGTGTTCGCACTTGTGAGGG<br>CT	421(874-1295)
Actb	https://www	GGGAATGGGTCAGAAGGACTCCTATGTGGGTGACGA<br>G<br>GCCCAGAGCAAGAGAGGTATCCTGACCCTGAAGTACC<br>CCATTGAACATGGCATTGTTACCAACTGGGACG<br>ACATGGAGAAGATCTGGCACCACACCTTCTACAATGA<br>GCTGCGTGTGGCCCCTGAGGAGCACCCTGTGCT<br>GCTCACCGAGGCCCCCCTGAACCCTAAGGCCAACCGTG<br>AAAAGATGACCCAGATCATGTTTGAGACCTTC<br>AACACCCCAGCCATGTACGTAGCCATCCAGGCTGTGCT<br>GTCCCTGTATGCCTCTGGTCGTACCACAGGCA<br>TTGTGATGGACTCCGGAGA	336(244-580)
Sox12	https://www	CACGAGCGGAGAAAAATCATGGACCAGTGGCCCGACA<br>TGCACAACGCTGAGATCTC<br>CAAGCGCCTGGGCCGCCGCTGGCAGCTGCTGCAGGACT<br>CGGAGAAGATCCCGTTCGTGCGGGAGGCGGAG<br>CGGCTGCGCCTCAAGCACATGGCGGACTACCCGGACTA<br>CAAGTACCGGCCTCGCAAGAAGAGCAAGGGGG<br>CGCCCGCCAAGGCGCGGCCCCGCCCCCCCGGAGGCGGC<br>GGTGGTGGCAGTCGGCTGAAACCCGGGCCACA<br>GCTGCCGGGCCGCGGGGGCCGCCGAGCGTCGGGAGGA<br>CCTCTGGGGGGCGGCGCGGCGGCGCCGGAGGAC<br>GACGACGAAGACGAAGAGGAG	357(505-862)

Map2	https://www	TGGTGGACGTGTGAAAATTGAGAGTGTAA<br>AACTGGATTTCAAGGAAAAGGCCCAAGCTA<br>AAGTT<br>GGCTCACTTGACAATGCTCACCACGTACCTG<br>GAGGTGGTAATGTGAAGATTGACAGCCAA<br>AAGTTGAACT<br>TCAGAGAGCATGCAAAGGCCCGGGTAGATC<br>ACGGGGCTGAGATCATCACACAGTCCCCAA<br>GCAGGTCCAG<br>CGTGGCATCACCCCGACGACTCAGCAACGTC<br>TCATCTTCTGGAAGCATCAACCTGCTCGAAT<br>CCCCTCAG<br>CTTGCCACTTTGGCTGAGGATGTCACTG	302(1617-1919))
Lmx1a			
Otx2			
Ascl1	https://www	GTGGCCGACAGCCAGCCCTCAGGGGGCGG<br>TCACAAGTCAGCGGCCAAGCAGGTCAAGCG<br>CCAGCGCTCGTCCTCTCCGGAACTGATGCGC<br>TGCAAACGCCGGCTCAACTTCAGCGGCTTCG<br>GCTACAGCCTGCCACAGCAGCAGCCGGCCG<br>CCGTGGCGCGCCGCAACGAGCGCGAGCGCA<br>ACCGGGTCAAGTTGGTCAACCTGGGTTTTG<br>CCACCCTCCGGGAGCATGTCCCCAACGGCGC<br>GGCCAACAAGAAGATGAGCAAGGTGGAGA<br>CGCTGCGCTCGGCGGTCGAGTACATCCGCG<br>CGCTGCAGCAGCTGCTGGACGAGCACGACG<br>CGGTGAGCGCTGCCTTTCAGGCGGGCGTCC<br>TGTCGCCCACCATCTCCCCCAACTACTCCAAC<br>GACTTG	397(762-1159)

Myc	https://www	GTCGCTACGTCCTTCTCCCCAAGGGAAGACG<br>ATGACGGCGGCGGTGGCAACTTCTCCACCG<br>CCGATCAGCTGGAGATGATGACCGAGTTAC<br>TTGGAGGAGACATGGTGAACCAGAGCTTCA<br>TCTGCGATCCTGACGACGAGACCTTCATCAA<br>GAACATCATCATCCAGGACTGTATGTGGAG<br>CGGTTTCTCAGCCGCTGCCAAGCTGGTCTCG<br>GAGAAGCTGGCCTCCTACCAGGCTGCGCGC<br>AAAGACAGCACCAGCCTGAGCCCCGCCCGC<br>GGGCACAGCGTCTGCTCCACCTCCAGCCTGT<br>ACCTGCAGGACCTCACCGCCGCCGCGTCCGA<br>GTGCATTGACCCCTCAGTGGTCTTTCCCTACC<br>CGCTCAACGACAGCAGCTCGCCCAAATCCTG<br>TACCTCGTCCGATTCCACGGCCT	321(855-1276)
Nanog	https://www	CCCACAGTTTGCCTAGTTCTGAGGAAGCATC<br>GAATTCTGGGAACGCCTCATCAATGCCTGCA<br>GTTTTTCATCCCGAGAACTATTCTTGCTTACA<br>AGGGTCTGCTACTGAGATGCTCTGCACAGA<br>GGCTGCCTCTCCTCGCCCTTCCTCTGAAGACC<br>TGCCTCTTCAAGGCAGCCCTGATTCTTCTACC<br>AGTCCCAAACAAAAGCTCTCAAGTCCTGAGG<br>CTGACAAGGGCCCTGAGGAGGAGGAGAAC<br>AAGGTCCTTGCCAGGAAGCAGAAGATGCG<br>GACTGTGTTCTCTCAGGCCCAGCTGTGTGCA<br>CTCAAGGACAGGTTTCAGAAGCAGAAGTAC<br>CTCAGCCTCCAGCAGATGCAAGAACTCTCCT<br>CCATTCTGAACCTGAGCTATAAGCAGGTTAA	400(600-1000)

sox2	https://www	CAAGCGGCTGCGCGCTCTGCACATGAAGGA<br>GCACCCGGATTATAAATACCGGCCGCGGCG<br>GAAAACCAAGACGCTCATGAAGAAGGATAA<br>GTACACGCTTCCCGGAGGCTTGCTGGCCCCC<br>GGCGGGAACAGCATGGCGAGCGGGGTTG<br>GGGTGGGCGCCGGCCTGGGTGCGGGCGT<br>GAACCAGCGCATGGACAGCTACGCGCACAT<br>GAACGGCTGGAGCAACGGCAGCTACAGCAT<br>GATGCAGGAGCAGCTGGGCTACCCGCAGCA<br>CCCGGGCCTCAACGCTCACGGCGCGGCACA<br>GATGCAACCGATGCACCGCTACGACGTCAG<br>CGCCCTGCAGTACAACTCCATGACCAGCTCG<br>CAGACCTACATGAACGGCTCGCCCACCTACA<br>GCATGTCCTACTCGCAGCAGGGCACCCCCGG<br>TATGGCGC	422(714-1136)
Klf4	https://www	CCTCCTAGCCCGGAGGGAGACCGAGGAGTT<br>CAACGACCTCCTGGACCTAGACTTTATCCTTT<br>CCAACTCGCTAACCCACCAGGAATCGGTGGC<br>CGCCACCGTGACCACCTCGGCGTCAGCTTCA<br>TCCTCGTCTTCCCCGGCGAGCAGCGGCCCTG<br>CCAGCGCGCCCTCCACCTGCAGCTTCAGCTAT<br>CCGATCCGGGCCGGGGGTGACCCGGGCGT<br>GGCTGCCAGCAACACAGGTGGAGGGCTCCT<br>CTACAGCCGAGAATCTGCGCCACCTCCCACG<br>GCCCCCTTCAACCTGGCGGACATCAATGACG<br>TGAGCCCCTCGGGCGGCTTCGTGGCTGAGC<br>TCCTGCGGCCGGAGTTGGACCCAGTATACA<br>TTCCGCCACAGCAGCCTCAGCCGCCAGGTGG<br>CGGGCTGATGGGCAAGTT	416(865-1281)

Shh	https://www	AAAAGCTGACCCCTTTAGCCTACAAGCAGTT<br>TATTCCCAA<br>CGTAGCCGAGAAGACCCTAGGGGCCAGCG<br>GCAGATATGAAGGGAAGATCACAAGAAAC<br>TCCGAACGATTT<br>AAGGAACTCACCCCCAATTACAACCCCGACA<br>TCATATTTAAGGATGAGGAAAACACGGGA<br>GCAGACCGGC<br>TGATGACTCAGAGGTGCAAAGACAAGTTAA<br>ATGCCTTGGCCATCTCTGTGATGAACCAGTG<br>GCCTGGAGT<br>GAAGCTGCGAGTGACCGAGGGCTGGGATG<br>AGGACGGCCATCATTCAGAGGAGTCTCTAC<br>ACTATGAGGGT<br>CGAGCAGTGGACATCACCACGTCCGACCGG<br>GACCGCAGCAAGTACGGCATGCTGGCTCGC<br>CTGGCTGTGG<br>AAGCAGGTTTCGACTGGGTCTACTATGAAT<br>CCAAAGCTCACATCCACTG	439(451-890)
Netrin1	https://www	GCTG<br>CAGATTCAACATGGAGCTCTATAAGCTATCA<br>GGGCGCAAGAGCGGGGGAGTCTGTCTCAA<br>CTGCCGCCAC<br>AACACTGCGGGCCGCCACTGCCACTACTGCA<br>AGGAGGGCTTCTACCGAGACATGGGCAAG<br>CCTATCACCC<br>ACCGGAAGGCTTGCAAAGCCTGTGATTGCC<br>ACCCAGTGGGTGCTGCTGGCAAGACCTGCA<br>ATCAAACCAC<br>TGGCCAATGTCCCTGCAAGGACGGCGTGAC<br>GGGCATCACCTGCAACCGATGTGCCAAAGG<br>CTACCAGCAG<br>AGCCGCTCCCCCATCGCCCCTTGCATCAAGAT<br>TCCTGTGGCGCCACCCACCACTGCAGCCAGC<br>AGCGTGG<br>AGGAACCGGAAGACTGTGACTCCTATTGCA<br>AGGCCTCCAAAGGCAAGCTGAAGATGAACA<br>TGAAGAAATA<br>CTGCAGGAAGGACTATGCTGTCCAG	449(1326-1775)

Dcc	https://www	TTCTTCAGCGCAGCAGAGGAAGAAACGGGC<br>CACACACAGTGTGAGCAAGAGGAAGGGCA<br>GTCAGAA<br>GGACCTCCGGCCTCCCGATCTTTGGATACAT<br>CATGAAGAAATGGAAATGAAAAATATCGA<br>GAAGCCTACG<br>GGAACCGACCCTGCAGGAAGAGACTCCCCC<br>ATCCAGAGCTGCCAAGATCTCACACCAGTCA<br>GCCATAGCC<br>AGTCAGAAACCCAGATGGGAAGCAAAAGT<br>GCCTCTCATTCAGGTCAGGACACTGAGGAC<br>GCAGGCAGCTC<br>CATGTCCACTTTGGAACGGTCCCTGGCAGCA<br>CGCCGGGCCACCAGGGCCAAGCTCATGATT<br>CCCATGGAG<br>GCCCAGTCCAGTAATCCTGCTGTTGTGAGTG<br>CCATCCCTGTACCAACACTAGAAAGTGCCCA<br>GTATCCAG<br>GAATCCTCCCATCTCCCACATGTGGATACCCG<br>CATCCACAGTTCACTCTCCGGCCAGTACCATT<br>CCCAAC<br>GCTGTCTGTGGACCGAGGTTTTGGAGCAG<br>GAAGAACTCAGTCTGTGAGTGAAGGACCAA<br>CCACGCAACAG<br>CAACCCATGCTGCCCCCAGCTCAGCCCGAAC<br>ATCCGAGCAGTGAAGAAGCACCCAGTAGAA	706(3995-4701)

Unc5b	https://www	GTACTCGGACCTGCACCAATCCAGCCCCACTC<br>AATGGAG<br>GCGCCTTCTGTGAGGGACAGGCCTTCCAGA<br>AGACAGCTTGCACCACCGTGTGCCCAGTGG<br>ATGGAGCGTG<br>GACCGAGTGGAGCAAGTGGTCTGCCTGCA<br>GCACAGAGTGTGCGCACTGGCGCAGCCGCG<br>AGTGCATGGCA<br>CCGCCACCCCAGAACGGAGGCCGTGACTGC<br>AGCGGGACGCTACTTGACTCCAAGAACTGC<br>ACTGATGGGC<br>TGTGCGTGCTGAATCAGAGAACTCTAAACG<br>ACCCTAAAAGCCACCCCCTGGAGACATCGGG<br>AGATGTGGC<br>ACTGTACGCAGGCCTTGTGGTGGCCGTCTTT<br>GTGGTGGTAGCGGTTCTCAT	370(1221-1591)
Unc5d	https://www	CGGAGTACCATGGCAAGA<br>ATCACTCGGGGACTTTCCCCCATGGAAACAA<br>CCGCGGATTCAGTACAATACATCCCAGAAAC<br>AAAACGCC<br>GTACATCCAAAATCTGTCATCACTGCCGACA<br>AGGACAGAGCTGAGGACAACTGGTGTCTTT<br>GGCCATTTA<br>GGGGGACGCTTAGTAATGCCAAATACAGG<br>GGTGAGTCTACTCATACCACATGGTGCCATC<br>CCAGAGGAGA<br>ATTCTTGGGAGATTTATATGTCAATCAACCA<br>AGGTGAACCGAGCCTGCAGTCAGATGGATC<br>TGAGGTTCT<br>CCTGAGTCCTGAAGTCACCTGTGGGCCTCCA<br>GATATGCTTGTCACAACTCCCTTTGCGCTGA<br>CCATCCCT<br>CACTGTGCAGACGTCAGTTCAGAGCACTGG<br>A	399(1803-2202)

Hnrnpa0	https://www	CCTCAATGTGCAGACGAGTGAGTCGGGGCT<br>GCGCGGCCACTTCGAGGCCTTCGGGACGCT<br>GACGGACTG<br>CGTGGTGGTGGTGAACCCCCAGACGAAGC<br>GCTCCCGCTGCTTCGGCTTCGTGACCTACTC<br>GAACGTGGAG<br>GAGGCGGATGCCGCCATGGCCGCGTCGCCG<br>CACGCGGTGGACGGCAACACGGTGGAGCT<br>GAAGCGCGCCG<br>TGTCGCGGGAGGATTCGGCGCGGCCCGGG<br>GCGCACGCCAAGGTGAAGAAGCTGTTCGTG<br>GGCGGCCTCAA<br>GGGCGACGTGGCGGAGGGCGACCTGATCG<br>AGCACTTCTCGCAGTTCGGCGCGGTGGAGA<br>AGGCGGAGATC<br>ATTGCCGACAAGCAGTCGGGCAAGAAGCGC<br>GGCTTCGGCTTCGTCTACTTC	400(282-682)
Oct4	https://www	gtgatgggtc agcagggctg gagccgggct<br>gggtggatcc tcgaacctgg<br>ctaagcttccaagggcctcc aggtgggcct<br>ggaatcggac caggctcaga ggtattgggg<br>atctccccatgtccgcccgc atacgagttc<br>tgcggaggga tggcatactg tggacctcag<br>gttggactgggcctagtccc ccaagttggc<br>gtggagactt tgcagcctga gggccaggca<br>ggagcacgagtggaaagcaa ctcagaggga<br>acctcctctg agccctgtgc cgaccgcccc<br>aatgccgtgaagttggagaa ggtggaacca<br>actcccgagg agtcccagga catgaaagcc<br>ctgcagaaggagctagaaca gtttgccaag<br>ctgctgaagc agaagaggat caccttgggg<br>tacacccaggccgacgtggg	430(121-550)

UTR sequences	UTR size(bp) and location in mRNA
AAGTGTGTGTGCCGTGGATAGCCCCTTGGCTGCTCT	
CCTGCAGAGAGACATCGGACAGACCTTAATTCTTACTCA	
CTGCTGTGGCTGGAGAGTATAAGGAATGCTT	
TTTCTTTTTTTCTTTCTTTCTTTCTTTTTTTTTTTTAAGACA	
GCAGTCTTTTTTTTTAATTTAAAAAAAA	
AAAGATATATTAACAGTTTTAGAAGTCAGTAGAATAAA	416(3674-4070)
CCTCCTAAGTAAGTCTTATTTTTAACTGAGC	
ATTGACAGTATCTTAAAATGGTAACGTGGGCGTGGGC	
GTTGTGTGCATAGCAGTCTAGCCGGTTGGGTAC	
CCTGCTCCTGTACCTAGTTCACAGACTCGAGTGCATTTT	
TTTTTGGCGAGATTTCCATCTTTGAAGAAAC	
AAAAAAACAAAAACACCTGAACCAGCGTTCTCTTATTCT	
TTAAGCTGTGGAAATAATTTCCAGTTTCTAC	
ATTCTCGATATGCATCCTTATTAAAAAGATAATACGAAT	
GAAAGGCAGTGTGCTTAAAGTGTGCTTTGCA	
AATACATGTTATGAATGACTACGGTCACTGGGCAAATT	388(4341-4728)
TTGTGAGGCAGGTTTACAACACTACACGTTTTGAATAA	
GAAGGAA	
AGAGAAAAAAATAAAAACACCAATACCCAGATTTAAAA	
AAAAAAAAAAAGATCATAGTCTTAGGAGTTCA	
TGTAAACCATAGGAACTTCCTGCTTATCTCATGTTAGCT	
GTACCAGTCAGTGATTAAGTAGAACTACAAG	
TTGTATAGGCTTTATTGTTTATTGCTGGTTTATGACCTT	
AATAAAGTGTAATTATGTATTACCAGCAGGG	
TGTTTTTAACTGTGACTATTGTATAAAAACAAATCTTGA	
TATCCTTCAGAAGCACATGAAGTTTGCAAGT	
CTCCACCCTGCCCATTTTTGTAAAACTGCAGTCATCTTGG	
ACCTTTTAAACACAAAATTTTAAACTCAAC	
CAAGCTGTGATACGTGGAA	414(1916-2330)
GCCTCATGGTCAAGAAAGGAGGGGGAAAATCCAGCGT	
G	
CCCCATCTCCTACCCACCCCCTCTTTGTATTCTCTTTGTAT	
TTTTCCCCTTCTTAAAATTTCTTTTTCTG	
CAATGAAGACAGAAAGAAGGCTCTGGGGTGATGCGTT	
TGGCATTTGTGTTGAGCTTAGGGGAGCATTGGC	
ATGGAGAAACTCCACGCTGGCGAAGTCCCGGGCTGGG	
CTTTCTCCTCCTCCCCCCACCTTTTTTCCCCTT	
GTCCTTGCAGCTGGAGATGTGCTGGGAGTAGCAGGCC	
AGCCTCGGAAATGGACATGGGGACCTCGTGGAA	
GCCACAGCAACCTGGTTGGGGATGCAGAAGGAC	351(2553-2904)

AAACAAACCACAAATAAAAACTGTCGCTATTTCCTAACC
TGCAGGCAGAACCTGAAAGGGCATTTTGGCTCCGGGG
CATCCTGGATTTAGAAAACGGACAGCACACAGTACAGT
GGTATAAACTTTTTATTATCAGTTCAAAATCAGTTTGTT
GTTCAGAAGAAAGATTGCTAATGTATGATGGGAAATG
TTTGGCCATGCTTGCTTGTTGCAGTTAAGACAAATGTA
ACACAC 234(2399-2632)

GTGGCTGAGGACTTTGTACATTGTTTTGTTTTTTTTTTT
TTTTGGTTTT
GTCTTTTTTTAATAGTCATTCCAAGTATCCATGAAATAA
GTGGTTACAGGAAGTCCCTCACCCTCCCAAA
AGCCACCCCCACTCCTAAGAGGAGGATGGTCGCGTCCA
TGCCCTGAGTCCACCCCGGGGAAGGTGACAGC
ATTGCTTCTGTGTAAATTATGTACTGCAAAAATTTTTTT
AAATCTTCCGCCTTAATACTTCATTTTTGTT
TTTAATTTCTGAATGGCCCAGGTCTGAGGCCTCCCTTTT
TTTTGTCCCCCCAACTTGATGTATGAAGGCT
TTGGTCTCCC 339(1492-1831)

GGC
TCCTCCCTAAGTCCATCACCCCTTCCTATCACTCAAATCCA
GTCTCAGCAACCACAACTGCAGTATTCAG
TGGGAGAAACTGAAGCACGTTGTTGCTAGGGTTTAGG
ACAGGCATACCAGCAGCCGGGTGGCAAGAATGA
GGACCATGTCTAAACAAACTTGGCTTGAGGAGAGACC
AGGCCAGGGGACGGTGGCAGAGCTAGTAGTGGA
CTTGGGTGCTTTGCACCTCGGTCTGCCCTTGTGAGAAA
TGGGGTGGCACTGCTGTAGTTAGGAAGCTGTG
TTGAGATTAGGTTGCCAAGTCTGCTCCACTGGTCTCCCG
CTCCAGCCCCGCCCCTCCAGCCTCCCCATCC
TCCCTCTGCCCTCACTACCTGTATCTCACCGGCG 387(2308-2695)

ATTGCAGGAAGGAAAGAGCATGTAAGAAACACATTTT
TTAAAGTGTTATTTTGTATAAATGGGAAGAAA
GACGCAATTAAGTTATTGACACTTGGGACCTGGACGA
GTATATCAGAGTATGCCATTCCAATAAATTATT
GAACTACAAGCTAGATTTAAGGCATTTGAGCGTTGGTT
GAAGAAGTGGTGTCAAAGTGCATCTCTTAGGA
TTGATGCACTTTTGTTAGGATGGGCTTGTGTCTGATTA
GAATGTCAGTCGATTGGCTAGATTTATATCCA
CACAATCAGTTTCACACCCCCATTCCATCTGTTTGATACA
GTATTATAGATATAAATATATATATATATT
TCTCTGTGGCCATTTGTGA 368(2382-2750)

AGACAGACACTATATTAACTCCCAACCACTAACAGGCAG
GGCTGGAAGCGCGCATGTGCAAGTGCCTTCACCTCCCA
CTCTCTGTCAGAGCTGTCTTAGCCCCCTGAAACTGGGTT
GATGTCTTTCCTCAGTCACCCCCATTCCAGCGATCTATG
GACATTTGCCTCCATTGAAGCAACGTCAGTTCTCGGACA
GCCTTTCCCTCTCCTGGTGGCCTCCTCCCCAAACCCCACA
TCGCCCTCCCACGGTCTTTGCTTCTGTTTTCTTCATAGAA
TGCTTCCAATCTTTGTGAATTTTTTTATTATAAGAAAAA
AATCTATTTGTATCTATCCTAACCAGTTTGGGGATATAT
TAAGATATTTTTGTACATAAGAAAAAGAGAGAGA
385(1605-1990)

ACTGACCTAACTCGAGGAGGAGCTGGAATCTCTCGTGA
GAGTAAGGAGAACGGTTCCTTCTGACAGAACTGATGC
GCTGGAATTAAAATGCATGCTCAAAGCCTAACCTCACA
ACCTTGGCTGGGGCTTTGGGACTGTAAGCTTCAGCCAT
AATTTTAACTGCCTCAAACTTAAATAGTATAAAAGAACT
TTTTTTTATGCTTCCCATCTTTTTTCTTTTTCCTTTTAACA
GATTTGTATTTAATTGTTTTTTTAAAAAAATCTTAAAAT
CTATCCAATTTTCCCATGTAAATAGGGCCTTGAAATGTA
AATAACTTTAATAAAACGTTTATAACAGTTACAAAAGA 346(1947-2293)

GGAGACAGTG
AGGTGCATATACTCTCTCCTTCCCAAGAATAAGTGCTTG
AACACCCTTACCCACGCCCACCCACCCATGC
TAGTCTTTTTTCTTAGAAGCGTGGGTCTTGGTATACACT
GTGTCATTTTGAGGGGTGAGGTTTAAAAGTA
TATACAAAGTATAACGATATGGTGGCTACTCTCGAGGA
TGAGACAGAAGGACCAGGAGTTTGAGGGTAGC
TCAGATATGCAATAAGTTCAAGGCCAACCTGTACTATG
TTTAAATAGTAAGACAGCATCTCGATAAAATA
ATAAAACTAAAGTCTCAACAAAATAAAAGCTTTCACCTA
TTAAGGTGCTTGCTTGTCCTTGGAGTCCCCC
AAGAGTAACTGCTATGTTAATATCTGTAGAAAGATGTT
TATATTTGACTG

500(1741-2141)

GCAAGCAACTTTTGTACAGTATTTATCGAGATAAACAT
GGCAATCAAATGTCCATTGTTTATAAGCTGAGAATTTG
CCAATATTTTTCGAGGAAAGGGTTCTTGCTGGGTTTTG
ATTCTGCAGCTTAAATTTAGGACCGTTACAAACAAGGA
AGGAGTTTATTCGGATTTGAACATTTTAGTTTTAAAATT
GTACAAAAGGAAAACATGAGAGCAAGTACTGGCAAGA
CCGTTTTCGTGGTCTTGTTTAAGGCAAACGTTCTAGATT
GTACTAAATTTTTAACTTACTGTTAAAGGCAAAAAAAA
AATGTCCATGCAGGTTGATATCGTTGGTAATTTATAAT
AGCTTTTGTTCAATCCT 359(1820-2179)

CTAACCTTTCACACTGTCTTCCCACGAGGGGAGGAGCCC
AGCTGGCAAGCGCTACAATCATGGTCAAGTTCCCAGCA
AGTCAGCTTGTGAATGGATAATCAGGAGAAAGGAAG
AGTTCAAGAGACAAAACAGAAATACTAAAAACAAACAA
ACAAAAAAACAAACAAAAAAAAACAAGAAAAAAAAAATC
ACAGAACAGATGGGGTCTGATACTGGATGGATCTTCT
ATCATTCCAATACCAAATCCAACTTGAACATGCCCGGAC
TTACAAAATGCCAAGGGGTGACTGGAAGTTTGTGGAT
ATCAGGGTATACACTAAATCAGTGAGCTTGGGGGGAG
GGAAGACCAGGATTCCCTTGAATTGTGTTTCGATGATG
CAATACACACGTAAAGATCACCTTGTATGCTC 407(21115-2522)

AGCGACTGCGAAATAAGGAACTGATGGGAAAGCGCAC
GGAAGGAGACTTTTAATTATAAG
AATAATTCATAATAATAATAATAATGATAATAATAATAA
TAATAAGTAGGGCAGTCCAAAGTAGACTATA
AGGAAGCAAAAACCCCGGGGAGTTCTGTTGTTATGTTT
AGTTTATATATTTTTTTGAAATTTTTCGTTAT
TGTCTTATATGGGTTGTTTTTCTCCTCTCCTGGCTATTTA
TTTGTTTCGTATGAATAGATGTTTTAAAAA
TATGAACGGACCTTCAAGAGCCTTAACTAGTTTGTGTCT
TGGATAATTTATTATTGTGTGAACTGTACTC
ACAGTGAGGGAAAGATTATTTTGTGAGGCCAAGCAAC
CTGCTGAAAGTCTATTTTTCTACATGTCCCTTG
TCCTGCGTTTCAGAAGGCAAACCTC 435(1691-2126)

GAGAGTGG
GCAAGATAGGCTCACTAATGGGCCGTGGTTCACAGAC
AGATATTCCTGTGGACCAGAGCCATGCCATACC
CCAGGGGTATCAAAATTGTCTTTGTGGGGCCTTGCCCC
TGCCAAAAACTAAGCCAGCCTCACCTCTTGTG
TCAGCGGACTTCCTTCCCCTTTCCCTGATCTGGGTACACC
CCCCCGGCCTCCCTCCTGTGTCACCGATTC
TGCTCACACAGAATTGTAAATGTTTAGTTGTGACCATG
ACATATTGTTTGGGCCAGTGTTCCTTTCCAAT
GCATACTAATATATTATGGTTATTATATATGAATATATT
TAATGACATGGAGAAAGTTGTGGATTTTCTT
TCTTTTTCTTTCTTTTTTTTTTTAAAGTTTTTTTGTTGTTA
GAGTTGTAATGGACCCAGACGGAACTTGT
AACGTGGGCCCTACAT 442(5244-5686)

TCTGGACCATTCGGTTAAGGAACATCTGAAGCACTT
CAGAAGAAAACAGTATCCCATCCTATATGGACATCAGG
AGTATACGATGATGGTCATGAGGTCCCGAAGA
TGCATCTTCACTGCAAAGTCAAGGCTGAAGCTGCTAGC
ATAGTTCTGGGTCTTTGTCACTGCAGGGACCA
TGGTGTCATAACTAATGCCTACATTTTCCTTCAGTGAGA
CCTTTCATTTTCTGTGTAGGTCTAGTCTCAC
AAGTGCATTATTTCAGCCTGTACCATGCCATAACAAATC
AATACAAACTCACTATTTTTTTCAGCTACAG
AGCATCCATGGTAACTCTCTCACTCCATAGCACCAAAGG
ATTGGATATTTTCCTGACAGCATAAAAGAAA
ATAAGTGAATAAGCAAAAGGCAGTGAATGCT 417(5075-5492)

ATGTGTGTGTGGGTGTGGTCAATAAGAGACT
GCACAGGGAAAGGCTTTGCTATGTCAAGGCCTCAGTTT
CCCCAGCTGTGCAGGGAGCTGAGACAGGGTTT
TAGCCATGGCCCTGAGTTCTAAAGTCACAAGCAGAAGC
TGGGGCCTGGTGGCCTTCGTCCTCTCCGGAGC
CCCCTGCAGGTGCCCTTCTGTGGCCTCCTCTGGCTTTGG
AGGGTCCAGCTGTGGTTCTGGGAGACTGTTC
AGGTCTGTTTGGTGATTCCTGGTCTCGTGATGGCCAAA
ACTCTTACTGAGAGGGAGGGGGTGTTCCAGGG
AGGGCTCTCTCCCTGGGTGAGGCTGGGTGGCTCTGTG
TCTAAGCTGGCACTAGCCGGTCTGCACCCCTGT
GCCTCTAGCCATCATTTTACCCGCTGATCTCCCTACTGGG
GTCAGGAGATATGAAGGTAACTAAGTAGAC
AGCTGTATACCCACTCTTGACCACAGCGCATTCTGGGAT
GTTCCATGGGGCCAGTGGAAGGGATGAATTC
TTGTCCCTTTCAGCCCACGGGCAGTGACAGGGACTAGG
ATCACTGGTATTGATCCTATTGAGATGCTGAC
TTCCCTGAACTGCCCTTGACCAGCCTGAAAGCTTTGAAA
GTTGGGGCCTCTGTGAGGAGGCAGGTTTCAT
GGCCACAGACCCAGCAAGCATCTCTGCCCTGGTTTTCCT 700(5296-5996)

CACTGTGACCCCAAGCTTTTGCATGACTTTCACCTGTCAC
AGCTCAAATGCCCATCCTGGAAGAGGTTT
CCACATTACTCTGGGCGCTAAATTCGGAAGTGTCACTG
TCCTAGTGCAAGATAAAACAAAAATTAAAAGT
GTCCTTAAATACTTAAAAACATAAATTTTTTACAAAAAC
AATCCTACTCAATGCCAAATTTCTCAGTGCT
TGGAGACATTAAATCTATACTTTAAATATGAGTTTAAAT
TTTGCCACAAAAATGGTGAGATCCACAGGTT
TGCTAGACTACAGCAGCATTACGTTAATTTACAGTCTCT
ACCACTCCACATACACAGTCTGAGTAGTTTA
CATTTGCATTCTTTAATCCAAAAGGAAACTTGTTCTGTG
GTTTTAAATGCTACCTTCATTCACCAGTGCG
GGTAGAC 426(6022-6448)

ATAGCTGCAAGACGGTTGGTTAA
GAAAAACCCAACTTACTTGTTCAGGACAATTTCATCAGA
TTTAGCAGTTTGCCATCAGAATTCTGCAGAC
TTGAAAGGTCCACATTTACTTGTAGCTGAGACGGGCTT
CCCGGGAGTGTAGTTAACAGCCCGCATCAGTC
ACTTTTTCTACGTGAGATTTGAACATGTAAACTGCTATG
CATAGCTTGGGCAGTAGCCCCAAACTGCTCC
GGTAGCTACTGAAGCAGCTGTGTGTACTGGGGTTTTA
GGTGCAAGTTGTAAAACAAAATATCTGTATTCT
GCTTGGTTAACGTGTATTTGTAGCCCTTCATGCAATAG
AATTCAAGTTGTTGTTTATAAAAAAAAAACAA
AAACTGATAATAGAAGAAAATGCCTTCCTGGA 405(2420-2825)

cctggggatg ctgtgagcca aggcaaggga ggtagacaag
agaacctgga gctttggggttaaattcttt tactgaggag
ggattaaaag cacaacaggg gtggggggtg ggatggggaa
agaagctcag tgatgctgtt gatcaggagc ctggcctgtc
tgtcactcat cattttgttcttaaataaag actgggacac
acagtagata 210(1141-1350)
