# Supplementary material for: Distinct expression of select and transcriptome-wide isolated 3’UTRs suggests critical roles in development and transition states
Source: PLoS One. 2021 May 5;16(5):e0250669. doi: 10.1371/journal.pone.0250669 (PMC8099112; doi:10.1371/journal.pone.0250669)
Supplement: S1 File — (ZIP) [file pone.0250669.s001.zip › Ji et al Supp info Plos One 12-29-20.pdf]

Ji et al Supporting information

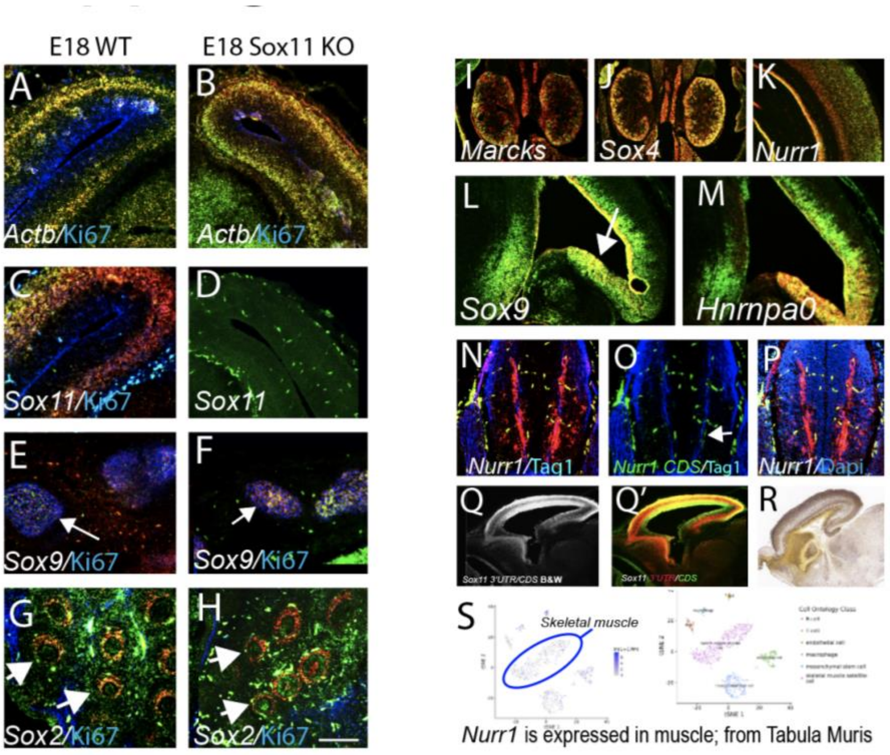

S1 Fig. As relates to Fig 1. (A-H) In situ hybridization for various probes in WT E18 and *Sox11* KO E18 tissues. All probes show signal in the *Sox11* KO animals except *Sox11* (green spots are background; compare C and D). (I, J) lower power views of Fig 1D,E showing entire kidney. (K) *Nurr1* in adult brain. (L-M) adjacent sections to Fig. 1 K-N, showing additional genes. (N-P) E11 spinal cord sections showing *Nurr1* 3'UTR/CDS (N) with low, but not "no signal" for the *Nurr1* CDS (O; green spots are background, white arrow points to signal). (P) same section with DAPI stain to show that background is not cellular and that many cells are *Nurr1* CDS and 3'UTR negative. (Q-R'), (Q,Q') *Sox11* 3'UTR/CDS in E15 sagittal brain in black and white (Q) and color (Q') compared to similar photo using the Allen brain Atlas CDS probe (R). With only one probe (Q,R) a meaningful pattern of *Sox11* is difficult to discern while the dual 3'UTR/CDS pattern suggests developmentally relevant expression (Q'). (S) Tabula muris data (https://tabula-muris.ds.czbiohub.org) showing widespread expression of *Nurr1* outside the brain and dopaminergic neurons. Highlighted is skeletal muscle. Scale bar, A-D; 500, E,F; 200, G,H; 400, I,J; 1500, K; 700, L,M; 300, N-P; 160,Q-R; 522 um.

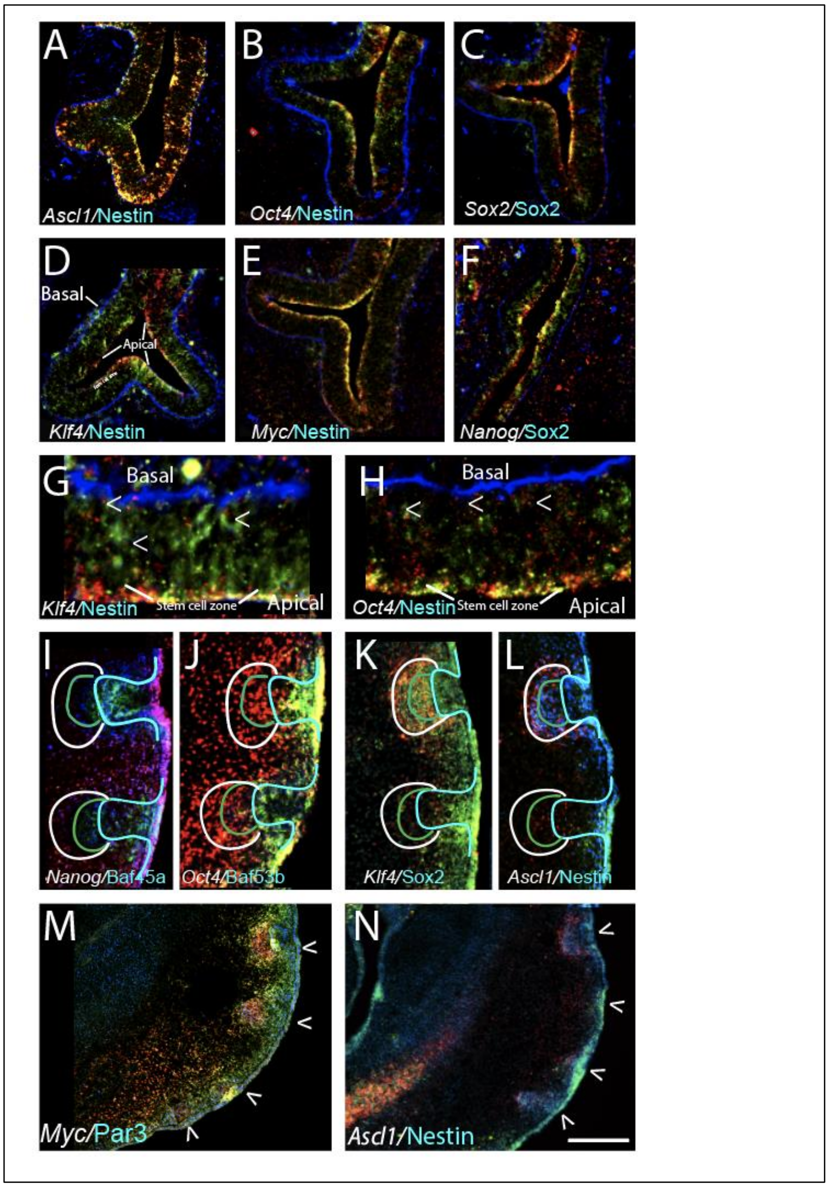

S2 Fig. As relates to 2. (A-F) Adjacent E12 OlfE sections to those in Fig. 2B,C, showing additional genes. All PpGs are expressed. (G,H) sections corresponding to Fig. 2D-G with additional PpG genes, Klf4 andOct4. (M,N) adjacent sections to Fig. 2 I,J with additional, indicated genes. Scale bar, A-C; 200, G-H; 150, I-L; 55, M,N; 350 um.

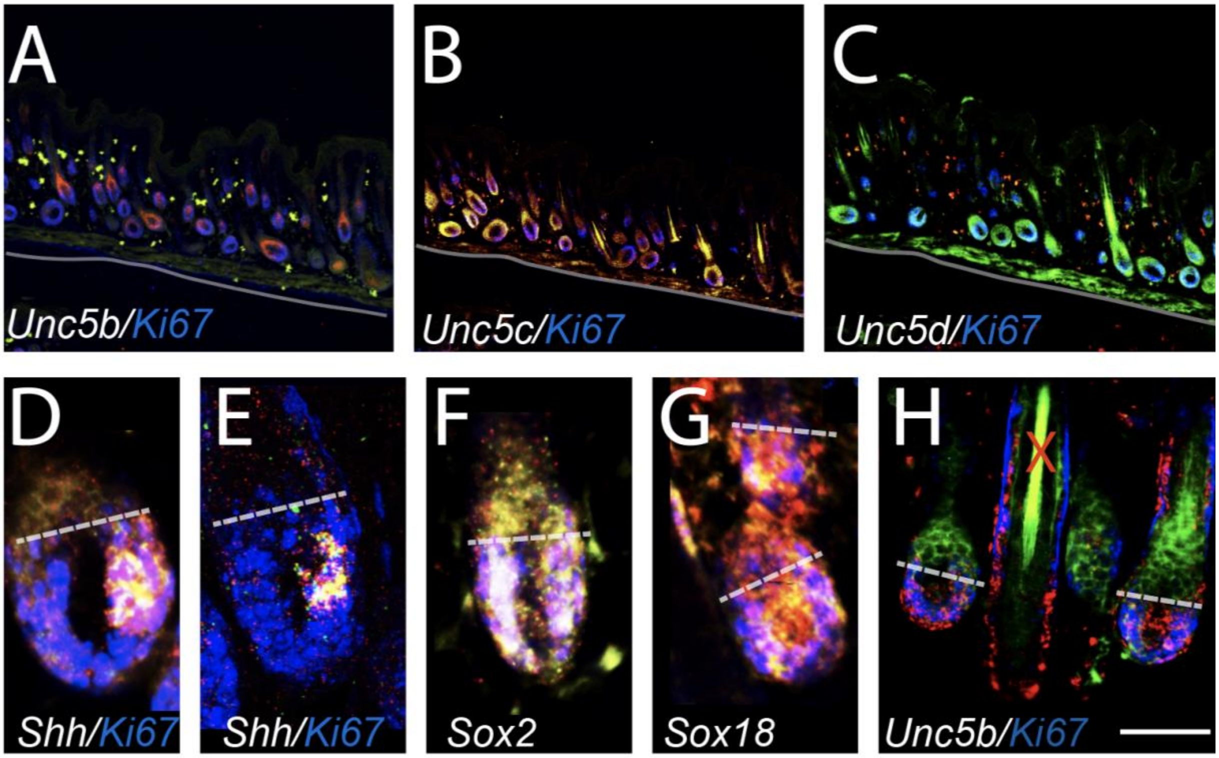

S3 Fig. As relates to Fig. 3. (A-C) Skin sections adjacent to Fig. 3D-F, showing additional genes as indicated. (D-G), adjacent sections to Fig. 3G-Q, showing additional genes as indicated. (D) *Shh* expression in Ki67+ cells (E) same as D but confocal image, note that pattern is identical in D,E. (H) same image as Fig. 2 M, M' but with hair shaft not cropped; note high auto-fluorescence in hair shaft, marked by red X. Scale bar A-C; 700, D-G; 85, H; 110 um.

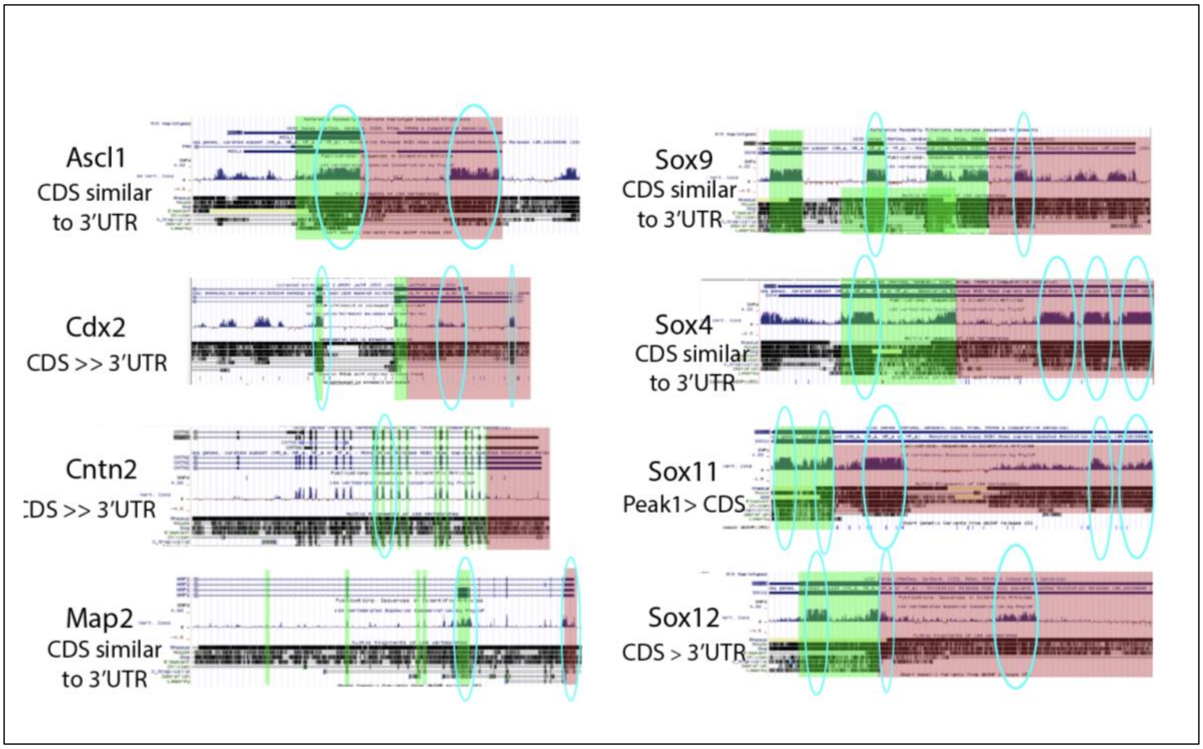

S4 Fig. As relates to Fig. 7. Conservation and ESTs. Some genes do not show high conservation within their 3'UTR across species, ie Cntn2 and Cdx2. Red shaded areas are 3'UTR, green CDS. Blue circles show regions of conservation.

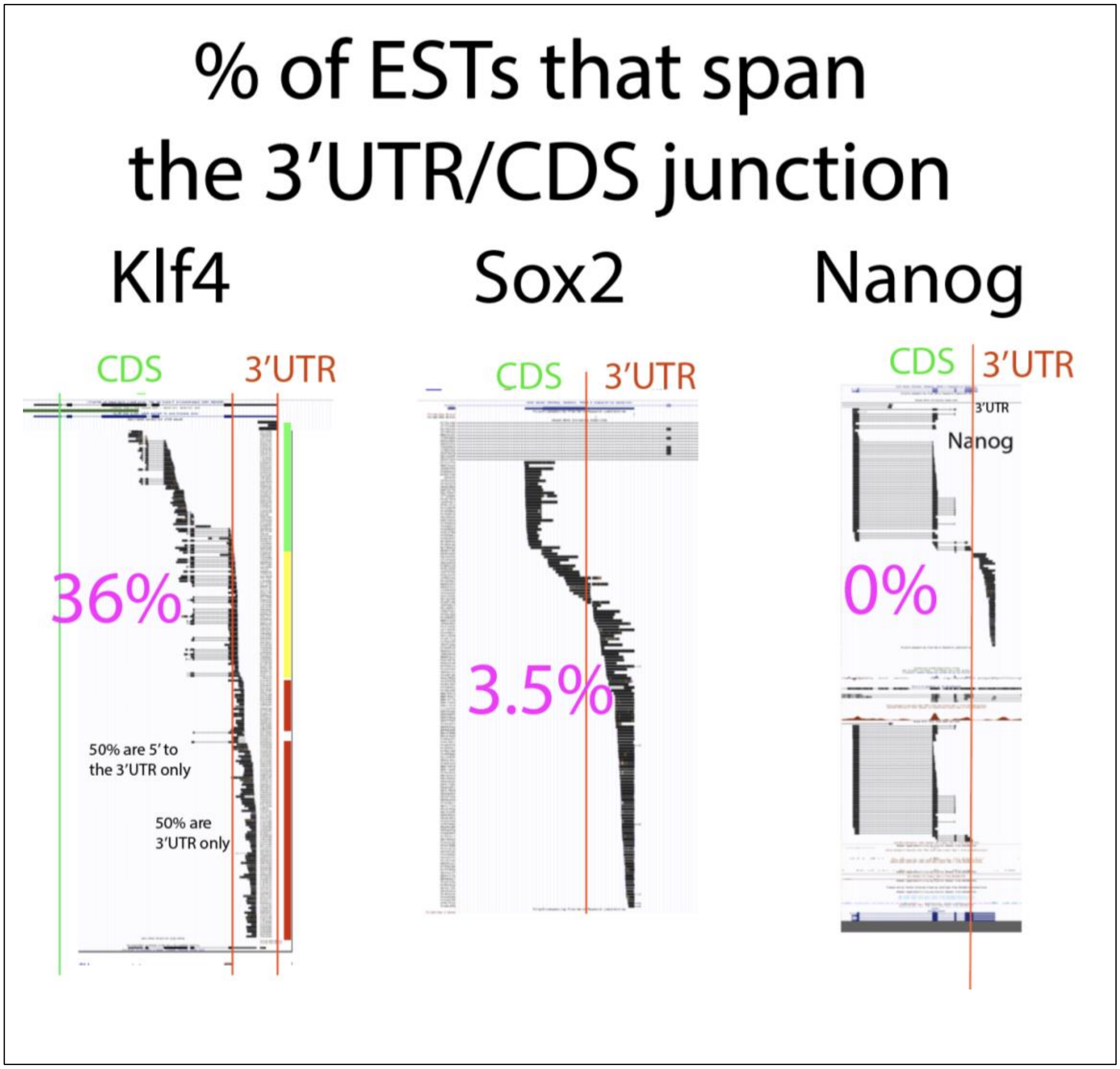

S5 Fig. As relates to Fig7. Percent of EST's that span the 3'UTR/CDS junction for three additional genes. *Klf4* shows a high preponderance of ESTs that span the 3'UTR/CDS junction, while the percent for *Sox2* is very low, and not one EST spans the junction for *Nanog*.

Ab	antigen	Dilution	Cat. #	Company
Tag1	Axon guidance molecule	1:500	AF4439	R&D Systems
Nestin	Neural stem	1:1000	Ab22035	Abcam
Sox2	Pluripotent gene	1:1000		R&D Systems
Baf45a	SWI/SNF complex Neural stem cells	1:30	N/A	Gift Dr. G. Crabtree
Baf53b	SWI/SNF complex; more differentiated cells	1:30		Gift Dr. G. Crabtree
MAP2	Structural protein	1:1000	NB300-213	Novus Biologicals
Foxa2 mouse<br><br>4C7 against chick	Transcription factor in DA neurons	1:50	N/A	Gift Susan Morton
Foxa2 K2 rabbit anti hnf3b	Transcription factor in DA neurons	1:10000	N/A	Gift Susan Morton
Ki67	Marker of active proliferation	1:1000		Abcam
Ddx6	P-body marker	1:100	Pa5-55012	Invitrogen
Xrn1	RNA degradation path; localized to P-bodies	1:100	Pa5 57110	Invitrogen
Rcd8	Mrna decapping P-body marker	1:50	Pa530485	Invitrogen

Lamin	Nuclear membrane	1:100	33-2100	Invitrogen

Supp. Table 1. Antibody list; companies, catalog number and dilution for antibodies used in these studies.

Supp. Table 2. In situ probe sequences
