# Supplementary material for: Distinct expression of select and transcriptome-wide isolated 3’UTRs suggests critical roles in development and transition states
Source: PLoS One. 2021 May 5;16(5):e0250669. doi: 10.1371/journal.pone.0250669 (PMC8099112; doi:10.1371/journal.pone.0250669)
Supplement: S1 File — (ZIP) [file pone.0250669.s001.zip › S3_Table.docx]

S3 Table. Raw sequencing reads acquired from GEO and ENCODE are as follows.

| **sample** | **Accession ID** |
| --- | --- |
| mESC | ENCSR000CWC |
| E11 forebrain | ENCSR160IIN |
| E15 midbrain | ENCSR557RMA |
| E15 heart | ENCSR597UZW |
| P0 hindbrain | ENCSR017JEG |
| adult cortex | ENCSR000BZS |
| P0 midbrain | ENCSR719NAJ |
| P0 skeletal muscle | ENCSR946HWC |
| adult heart | ENCSR000BYQ. |
| heart E10.5 | ENCSR049UJU |
| heart E15.5 | ENCSR597UZW |
| heart P0 | ENCSR526SEX |
| hindbrain E10.5 | ENCSR943LKA |
| hindbrain E15.5 | ENCSR401BSG |
| hindbrain P0 | ENCSR017JEG |
| 18 adult tissue | PRJNA375882 |
